# Visualization of unstained homo/heterogeneous DNA nanostructures by low-voltage scanning transmission electron microscopy

**DOI:** 10.1038/s41598-020-61751-3

**Published:** 2020-03-17

**Authors:** Geun Won Gang, Jihoon Shin, Young Heon Kim, Tai Hwan Ha, Takashi Ogawa

**Affiliations:** 1 Advanced Instrumentation Institute, Korea Research Institute of Standards and Science (KRISS), Daejeon, 34113 Republic of Korea; 2 Department of Physics, Chungnam National University, Daejeon, 34134 Republic of Korea; 3 BioNanotechnology Research Center, Korea Research Institute of Bioscience and Biotechnology (KRIBB), Daejeon, 34141 Republic of Korea; 4 Division of Industrial Metrology, Korea Research Institute of Standards and Science (KRISS), Daejeon, 34113 Republic of Korea; 5 Graduate School of Analytical Science and Technology (GRAST), Chungnam National University, Daejeon, 34134 Republic of Korea

**Keywords:** Single-molecule biophysics, DNA nanostructures, Organizing materials with DNA, Scanning electron microscopy, Transmission electron microscopy

## Abstract

Three-dimensional (3D) homo/heterogeneous DNA nanostructures were studied with low-voltage scanning transmission electron microscopy (LV-STEM). Four types of 3D DNA nanostructures were designed and fabricated by the origami method including newly proposed protocols. The low-energy electron probe and optimized dark-field STEM detector enabled individual unstained DNA nanostructures to be clearly imaged by the single acquisition without the averaging process. For the vertically stacked double structures, assembled through modified single-stranded domains, and the structures containing a square opening (i.e., a hole) in the center, the LV-STEM successfully reveals the vertical information of these 3D structures as the contrast differences compared to the reference. For the heterogeneous structures, the LV-STEM visualized both regions of the functionalized gold nanoparticles and the DNA base structure with distinct contrasts. This study introduces a straightforward method to fabricate stackable DNA nanostructures or nanoparticles by replacing a relatively small number of incumbent DNA strands, which could realize the simple and sophisticated fabrication of higher-order 3D DNA homo/hetero nanostructures. Together with these design techniques of DNA nanostructures, this study has demonstrated that the LV-STEM is the swift and simple method for visualizing the 3D DNA nanostructures and certifying the fabricated products as the specified design, which is applicable to various research fields on soft materials including DNA nanotechnology.

## Introduction

DNA nanotechnology, a branch of structural nanotechnology, enables the fabrication of nanostructures with various shapes and structures in all dimensions based on the designability and structural stability of the DNA duplex^1^. Because of the geometrical freedom that they offer for real-world applications such as nano-cages (Chandrasekaran, *et al*.)^2^ for cellular delivery (Schaffert, *et al*.)^3^, construction of biomimetic systems (Burns, *et al*.)^4^, and DNA nano-molds for inorganic fabrication (Helmi, *et al*.)^5^, over the past decade, numerous studies have been conducted on three-dimensional (3D) shapes using DNA origami folding methods^6–8^ and lattice fabrication from DNA motifs^9–11^.

Among 3D DNA nanostructures, origami structures, which would fold into two-dimensional (2D) and even 3D structures from a single long scaffold DNA strand with staple strands, have attracted attention for their structural stability and well-defined size at the molecular level. As interest in DNA nanostructures grows, their observation and measurement methods are also becoming important because of the necessity of evaluating whether synthesized specimens satisfy the intended design. The methods should be robust and straightforward and it would be preferable if the methods are rapid and simple as possible. Additionally, emerging research trends in 3D nanostructures^12–14^ require evaluation methods for not only 2D geometries on the horizontal plane but also 3D nanostructures with a degree of freedom in the vertical direction.

In previous studies, atomic force microscopy (AFM) has been the main technique used to measure 2D DNA nanostructures (algorithmic self-assembly of DNA tiles or DNA origami) in ambient environments or even in environments specifically designed to aid the observation of well-preserved geometries^15–18^; AFM is, in fact, the most compelling methodology to reveal geometrical shapes of soft DNA nanostructures in solution phase. However, AFM measurement struggles to measure the exact inner morphologies of 3D DNA origami structures, which originates from the inherent principle of AFM via tip-sample interactions^19,20^. At this point, electron microscopy (EM) measurement rather stands out as a minimally invasive tool that can see the inner features of 3D DNA structures.

As an alternative method or complement, transmission electron microscopy (TEM) is commonly used to observe and analyze various types of 3D DNA origami structures. With the TEM under high beam energy conditions typically from 100 to 300 keV, the designed DNA origami structures in various sizes from several nanometers to micrometers can be visualized and analyzed with sufficiently high resolution^6–8,13^. However, through the TEM observation, the specimen can be damaged by several effects such as surface charge interaction, dehydration, and electron beam exposure, and staining process^21^. Among these damages, the staining process is an unavoidable step to observe the DNA origami structures for the TEM imaging with sufficient quality. The specimens are usually stained with heavy materials such as uranium to enhance the signals under the high-energy conditions of conventional TEM (100–300 keV).

However, the staining process causes irreversible structural damages to the specimen such as deterioration of its biological functions and intra-strand cross-linking^22^. In addition, the staining prevents the acquisition of information on the vertical direction of the DNA structures because of the non-uniform coverage or thickness of the DNA structures. On occasions, the specimens were positively stained without intention under typical negative staining protocol^23,24^. Staining material such as uranyl acetate is toxic and environmentally hazardous and also causes additional side effects, for example, undesirable interaction with the specimen and structural artifacts in images^25–27^.

For those reasons, direct imaging of unstained DNA nanostructures from a single acquisition has been explored. Kabiri *et al*.^28^ have reported the undesirable distortion occurred in 2D DNA nanostructures supported on graphene films through observation of the nanostructures both with and without staining using aberration-corrected scanning transmission electron microscopy (STEM) at 300 keV. Compared with the stained DNA nanostructures on conventional amorphous carbon films, which showed clearer structures with sharp edges, the unstained ones exhibited unclear edges and were buried under the background patterns of the graphene films.

Cryogenic electron microscopy (Cryo-EM) might be another option for the direct imaging of DNA nanostructures and the technique has successfully visualized various types of DNA complexes and realized the structural analysis in a high-resolution of sub-nanometer^12,29–31^. However, this method usually requires multiple images of the specimens, amounting to the thousands, and additional image processing techniques including class averaging are also necessary, even with those sophisticated treatments, the observation of individual DNA nanostructures from single image acquisitions is still difficult at many cases. Moreover, the ice-embedded condition itself could prevent the DNA nanostructures from adopting physiological and natural states or structures, and thus hampers one to observe and/or analyze biological functions, when using electron spectroscopy^32^.

For another attempt to observe soft biological samples intact, the effectiveness of low-voltage STEM in scanning electron microscopy (LV-STEM in SEM) has been recently demonstrated on various scales from micro- to nanometers, such as a single cell^33^ and a bacteriophage^34^. Even though the observation causes several damages similarly to TEM, that is, surface charge interaction, dehydration, and electron beam exposure, the imaging technique of LV-STEM, which operates typically under low-energy conditions of 30 keV, enabled observation of unstained biological materials with high contrast due to the strong interactions between low-energy electrons and the specimen. In our previous study^35^, the twisted structures of insulin amyloid fibrils were clearly observed with a specially designed dark-field (DF) STEM detector that efficiently collected signals from light-element materials.

In this study, we designed and fabricated vertically stackable DNA nanostructures using scaffold DNAs, with stepwise adjustability of structural thickness, to demonstrate the image quality and capability of 3D information determination for unstained specimens using an LV-STEM system. Additionally, we fabricated a nano-square holed DNA nanostructure (nsh-DN) to reduce the thickness at the center of the DNA nanostructure, with the hole openings normal to the DNA helix direction. Then, we modified the nsh-DN by introducing single-stranded AuNP-capturing domains to fabricate a heterogeneous DNA nanostructure, wherein the nano-square holes were filled by DNA-functionalized gold nanoparticles (AuNPs) through DNA domain hybridization. This heterogeneous DNA nanostructure with AuNPs (h-DN) was used to reveal the full potential of our LV-STEM system, which provides a sufficient contrast of the DNA origami region to distinguish two different types of materials (i.e., DNA and AuNP).

Our imaging of these specimens shows that, although there is some loss of resolution compared with the conventional TEM method using staining, visualization of staining-free DNA nanostructures using LV-STEM enables the evaluation of designed structures with well-preserved biological function.

## Results and Discussion

### Synthesis of DNA nanostructures

We designed a 3D rectangular DNA nanostructure based on a previously reported structure^3^, henceforth denoted as the single DNA nanostructure (s-DN). Then, a doubly stacking DNA origami structure (d-DN) was fabricated by combining two s-DNs vertically. Meanwhile, another s-DN was redesigned to a nano-square holed DNA nanostructure (nsh-DN), which had an opening at the center in the normal direction to the DNA helix. A heterogeneous DNA nanostructure was assembled by hybridization between functionalized AuNPs and nsh-DN with four single-stranded AuNP-capturing domains at the four vertexes of the squared hole.

To fabricate the s-DNs, the structures were thermally annealed in a one-pot reaction, where the scaffold (p8064) was mixed with 10-fold excess of staple strands (156 staple strands were needed for each s-DN). The s-DNs had planar dimensions of 59.2 nm × 28.5 nm and were 7.4 nm in thickness.

**Figure 1:**
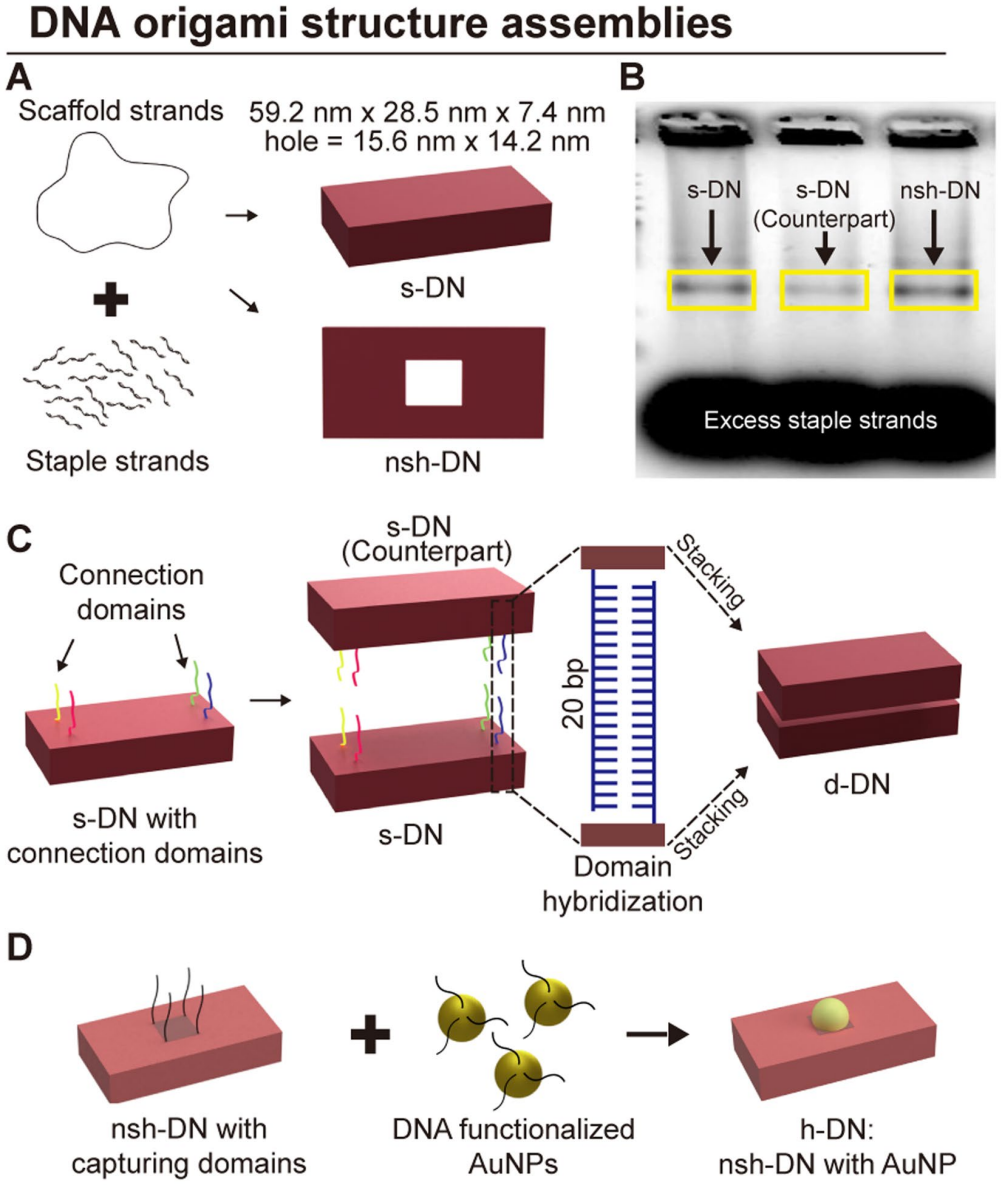
Design and synthesis of DNA origami nanostructures. (**A**) Schematics of the rectangular and nano-square holed DNA origami nanostructures. The DNA regions are represented in red with the dimensions of the structures. (**B**) Agarose gel electrophoresis data of the s-DN, counterpart of the s-DN, and nsh-DN. These structures were used for the fabrication of doubly stacking and heterogeneous DNA nanostructures. The clear and narrow bands in each yellow box, which are indicated by black arrows, show the high folding yield of the structures. The blurred bands indicate excessive staple strands that were removed by physical excision. (**C**) Schematic of the doubly stacking of a DNA nanostructure by combining two single DNA nanostructures via 4 pairs of hybridized DNA domains. Each individual domain has 20 nucleotides, as indicated in different colours for each pair, i.e., a domain hybridizes with a counterpart of the same colour. (**D**) Schematic of the self-assembly of the heterogeneous DNA nanostructure with a nano-square holed structure and a DNA functionalized gold nanoparticle (AuNP). The capturing domains of the nano-square hole and the AuNPs are shown in black and yellow, respectively.

The nsh-DNs were designed to form the same geometry as s-DN and had a nano-square hole of 15.6 nm × 14.2 nm. The nano-square holed structure was folded from an M13mp18 scaffold and 131 staple strands (Fig. 1A).

After a thermal annealing process, the accurate formation of the structures of the s-DN, the counterpart of the s-DN for forming the d-DN, and the nsh-DN was verified through agarose gel electrophoresis. To purify the structures, each band was physically excised and then the structures were extracted from the bands through spin column method (Fig. 1B).

We thus introduced a simple method to fabricate higher-order structures. The four incumbent staple strands were replaced by new four strands that have the same sequences of the corresponding incumbent staple strands with additional 20 nucleotides. Due to the difficulties of determining the exact location of multivalent target particles (e.g., DNA-AuNP conjugations) immobilization, multiple capturing sites provide better addressability for multivalent target particles^36^. For stacking the s-DNs, we have used this design method, and replaced the four existing staple strands with four new strands with single-stranded domains. By replacing eight different strands, s-DNs can be divided into s-DNs with capturing domains and their counterparts for fabricating d-DNs (Fig. 1C). Depending on the position of the replacement and providing four different complementary sets, the method gives anisotropic binding to d-DN with relatively very small structural modifications. Each s-DN with connection domains was assembled with its counterpart. These connections were made by combining the four single-stranded connection domains that made up the complementary bond. The single-stranded domain consisted of 20 unpaired nucleotides, which protruded perpendicularly to the surface of the origami structure. Figure 1C shows the fabrication of d-DN which is designed by the aforementioned method. Through the fabrication of d-DN, we were able to increase the thickness of the origami structure beyond the intrinsic volume limitation, by stacking one or two origami structures onto the 3D origami structure, guided by the connection strands.

To further probe the performance of the LV-STEM in SEM system using unstained substrates, AuNPs and nsh-DN were co-assembled into h-DN. We anticipated that the functionalized AuNPs could be captured by domains consisting of four single strands of the same sequence that could hybridize with the strands attached to the AuNPs. Figure 1D explains this process.

### The LV-STEM in SEM for the DNA nanostructure imaging

**Figure 2:**
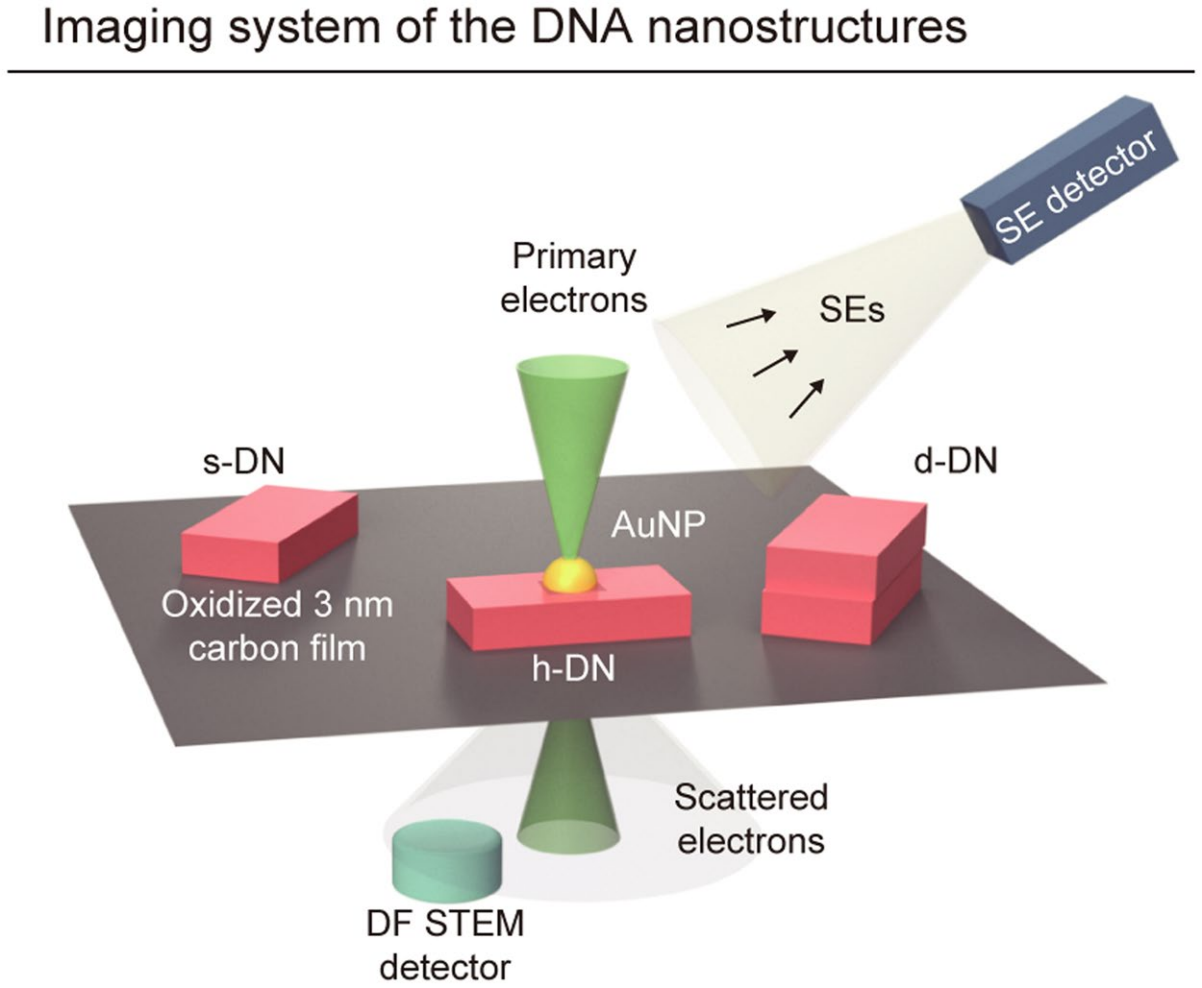
The imaging system using LV-STEM in SEM for observing 3D DNA nanostructures. The primary beam was focused on and scanned across each specimen. The DF-STEM detector collected scattered electrons by the interaction with the specimens, mainly at low angles, to acquire transmission images. The SE detector collected secondary electrons for surface imaging. Supporting substrates were carbon films with a thickness of 3 nm that were oxidized by plasma pretreatment.

Figure 2 shows the visualization method. The DNA specimens were deposited on amorphous carbon substrates with a thickness of 3 nm, which were exposed to an oxygen plasma to make their surfaces hydrophilic as a pretreatment (See Materials and Methods). To observe the unstained DNA nanostructures, we installed a homemade STEM detector into an in-lens cold field emission SEM (S-5000, Hitachi, Tokyo). This detector was specially designed to detect the electrons scattered at low angles from 15 to 55 mrad, allowing dark-field imaging, with high efficiency to enhance the image contrast from non-stained DNA nanostructures. Meanwhile, a conventional secondary electron (SE) detector of the SEM was simultaneously used to acquire complementary information of the specimen.

### Characterization of DNA structures

In the initial stages of this study, two kinds of structure (s-DN and nsh-DN), were first evaluated with AFM in liquid (See Figs S1 and S2, respectively, in Supporting Information section 2). The s-DNs showed a rectangular shape as expected, but the measured height was slightly low (~2.5 nm), which was originally designed to be 7.4 nm. For the nsh-DN, it was hard to observe the central opening of the specimens because of unintended side effects, namely the convolution effect which is making a protruding object wider and a sinking object narrower in a lateral direction, and deformation of the DNA origami structure during AFM measurement, manifesting the necessity of alternative observation methods. In addition, DNs were observed with a conventional TEM at 300 keV. (See Supporting Information section 3. Figure S3 shows TEM images of DNs with and without staining.) With the use of staining, DNs were barely observed with the TEM. Without staining, it was difficult to distinguish the DNA regions from the substrates because of its low contrast due to high energy conditions. The direct observation of the unstained DNs failed with the conventional TEM.

**Figure 3:**
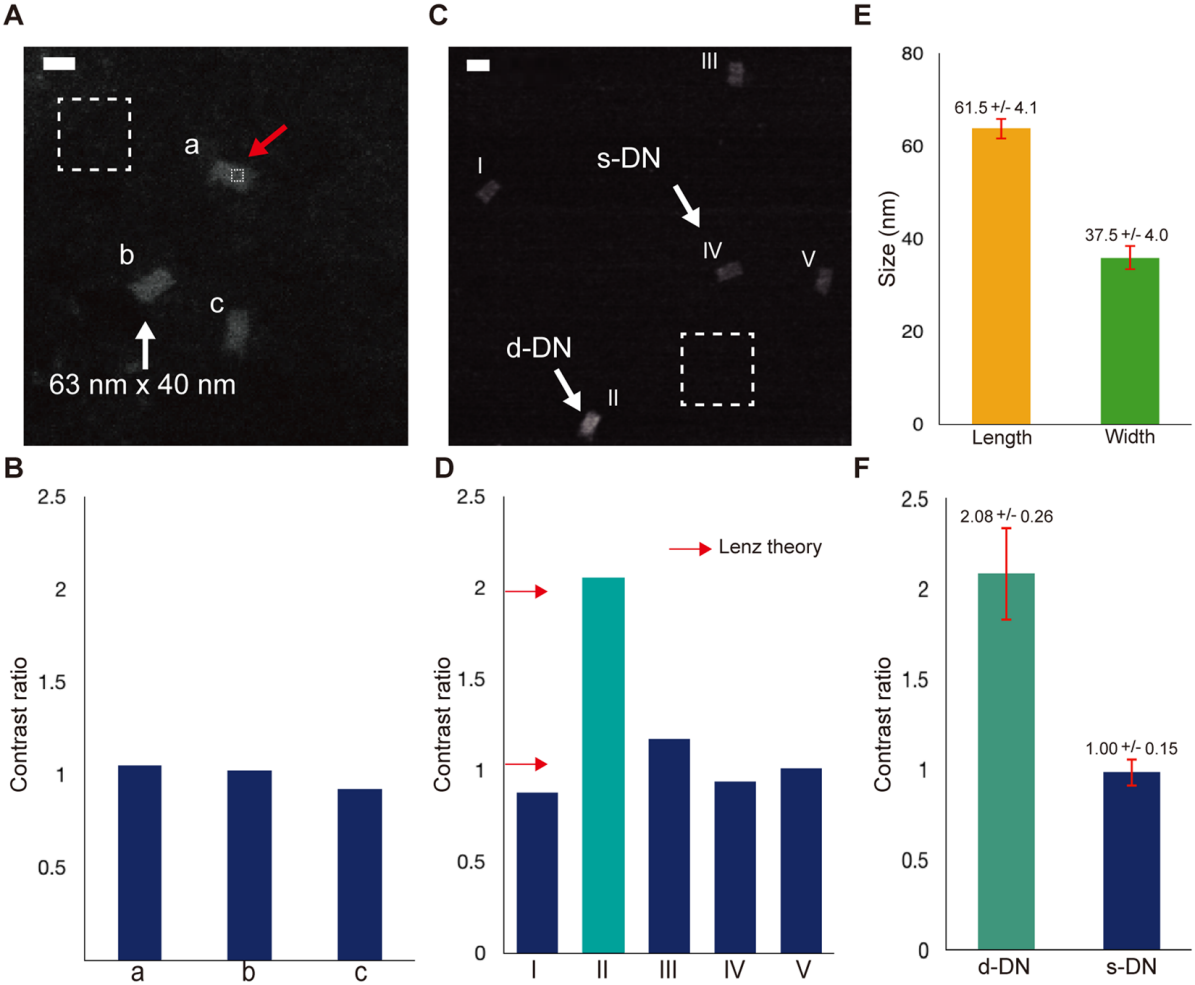
STEM observation and analysis of the s-DNs and d-DNs. (**A**) STEM image of the unstained s-DNs (a, b, and c). The three structures showed similar dimensions and contrast. For the contrast analysis, the signal intensities of the DNA origami and background were measured from the regions marked by smaller and larger dashed boxes in white, respectively. One of the small boxes for the DNA origami signal is indicated with a red arrow. Scale bars are 50 nm in all images. (**B**) Contrast ratios of three DNA structures (a, b, and c). The three structures all showed contrast ratios close to the value of 1. (**C**) STEM image of the d-DN (II) and four s-DNs (I, III–V). (**D**) Contrast ratios of the five DNA structures (I–V). The theoretical values calculated with Lenz’s theory are indicated by red arrows. The four s-DNs (I, III–V) in blue all showed similar contrast ratio values of approximately 1. The structure (II) in cyan showed twice the value compared with the s-DNs. The measured values are consistent with theoretical predictions. (**E**) The dimension of 110 sets s-DNs. The bars in yellow and green correspond to averaged values of width and length respectively. The error bars show the standard deviations. (**F**) Contrast ratios of 110 sets of s-DNs and 10 sets of d-DNs in cyan and blue, respectively. The error bars show the standard deviations.

Subsequently, the s-DNs on carbon film were observed under unstained conditions with the LV-STEM proposed in this study. Figure 3A shows three unstained s-DNs (a, b, c) with rectangular shapes in bright contrast. The dimensions of s-DN (b) in Fig. 3A were 63 nm × 40 nm, which was consistent with the designed values. The STEM images show clearer and sharper edges of the structures compared with the AFM images. With the LV-STEM, the DNA nanostructures can also be located and observed much faster than with AFM observation. The simple operational procedures were the same as for conventional SEM, that is, in terms of adjustment of focus and stigmators.

In STEM imaging mode, the contrast values of the image generally reflect the mass thickness of the specimen. For larger or thicker biomaterials, the TMV^37^ has been used as a reference to estimate the mass thickness of the biomaterials. For a nanometer-scale structure, the contrast of monolayer graphene can be used as a standard mass thickness to count the number of layers of multilayered graphene in a STEM image^38^. Comparing the contrasts of the target specimens to that of well-defined references, the thickness, that is, vertical information, of the specimens can be determined if the specimens and references have equivalent composition and density.

Likewise, three s-DNs was evaluated by the contrast analysis method; the contrast of DF-STEM images was assumed to be proportional to the mass thickness, (the multiplication product of the density and thickness of the specimen) (See Supporting Information section 4). Because the DNA origamis were assumed to have the same density over the whole structures, the contrast was proportional to the thickness. In this study, the contrast value of a DNA origami was defined as

1 $${\rm{Contrast}}={({\rm{I}}}_{{\rm{D}}}-{{\rm{I}}}_{{\rm{B}}}{)/{\rm{I}}}_{{\rm{B}}},$$

where *I*_D_ and *I*_B_ are the signal intensities of the DNA origami and the background, respectively. In the STEM images in Fig. 3, *I*_D_ and *I*_B_ were measured at the regions in the larger and smaller dashed white boxes, respectively.

Figure 3B shows the measured contrast values of the three DNA nanostructures (a, b, c) in Fig. 3A. For ease of comparison, the vertical axis is shown as a contrast ratio, where the contrast values were divided by their averaged value. The contrast ratios of the three DNA nanostructures were all close to 1, indicating that the thicknesses of the three s-DNs were equivalent.

To verify the reliability as the reference structure in the experiments, an additional set of 110 s-DNs were evaluated. (See Supporting Information section 6. The original LV-STEM images of the s-DNs were shown). The dimensions of the s-DNs were 61.5 ± 4.1 nm × 37.5 ± 4.0 nm in averaged values and standard deviations and the contrast ratio was 1.00 ± 0.15, as summarized in Fig. 3E and F, respectively. Those measured values are well consistent with the results in Fig. 3B, confirming that the s-DNs have not only the geometric dimensions as our design specification but also showed possibility as the references in the mass-thickness analysis.

Applying the same STEM imaging method to distinguish the d-DNs from the s-DNs, Fig. 3C shows five DNA structures (I-V) from d-DN samples. Among them, one d-DN (double layered DNA structure, II) in Fig. 3C was definitely observed with brighter contrast than the four s-DN structures (I, III, IV, V), which showed similar contrasts to one another.

Figure 3D gives thus-calculated contrast ratios of the five DNs (I-V); the contrast values of each DNs were divided by the averaged value of the four s-DNs (I, III, IV, V). In Fig. 3D, the contrast ratio of structure (II) was almost twice that of the other four structures. According to interpretation by analyses based on Lenz’s theory, the theoretically calculated contrast ratio of the d-DN was 1.94 times higher than that of the s-DN under the present experimental condition (See Fig. S4 in Supporting Information section 4). These theoretical values are indicated by red arrows in Fig. 3D and the agreement between theory and experiment confirms that the DNA structure (II) is the d-DN.

Furthermore, Fig. 3F summarizes the statistical data of additional 10 d-DN structures, demonstrating an averaged contrast ratio being close to 2. The difference in contrast ratios between the d-DNs and s-DNs was much larger than the standard deviations (shown in error bars), which statically confirms the identification of d-DN.

The results in Fig. 3 show that the LV-STEM can clearly visualize DNA origami structures in a rapid and simple manner. Together with the measurement of the dimensions in 3D, it is confirmed that our LV-STEM system can characterize multi-stacking DNA nanostructures.

Besides, Fig. S7 is an additional result of the LV-STEM imaging (See Supporting Information section 7). The single acquisition image shows three DNA origami structures with shape and dimension similar to the s-DN. A DNA structure with a defect can be distinguished intuitively from other normal structures with a higher resolution, which is adequate to verify the locally damaged region in the DNA structure. The result means that this analysis method is straightforward and does not require any specific prerequisite of the structural symmetry. The result also suggests the application of the LV-STEM with sufficient imaging capability to a process monitor, which certifies whether DNA nanostructures are fabricated as per the specified design.

### Analysis of the heterogeneous structure

**Figure 4:**
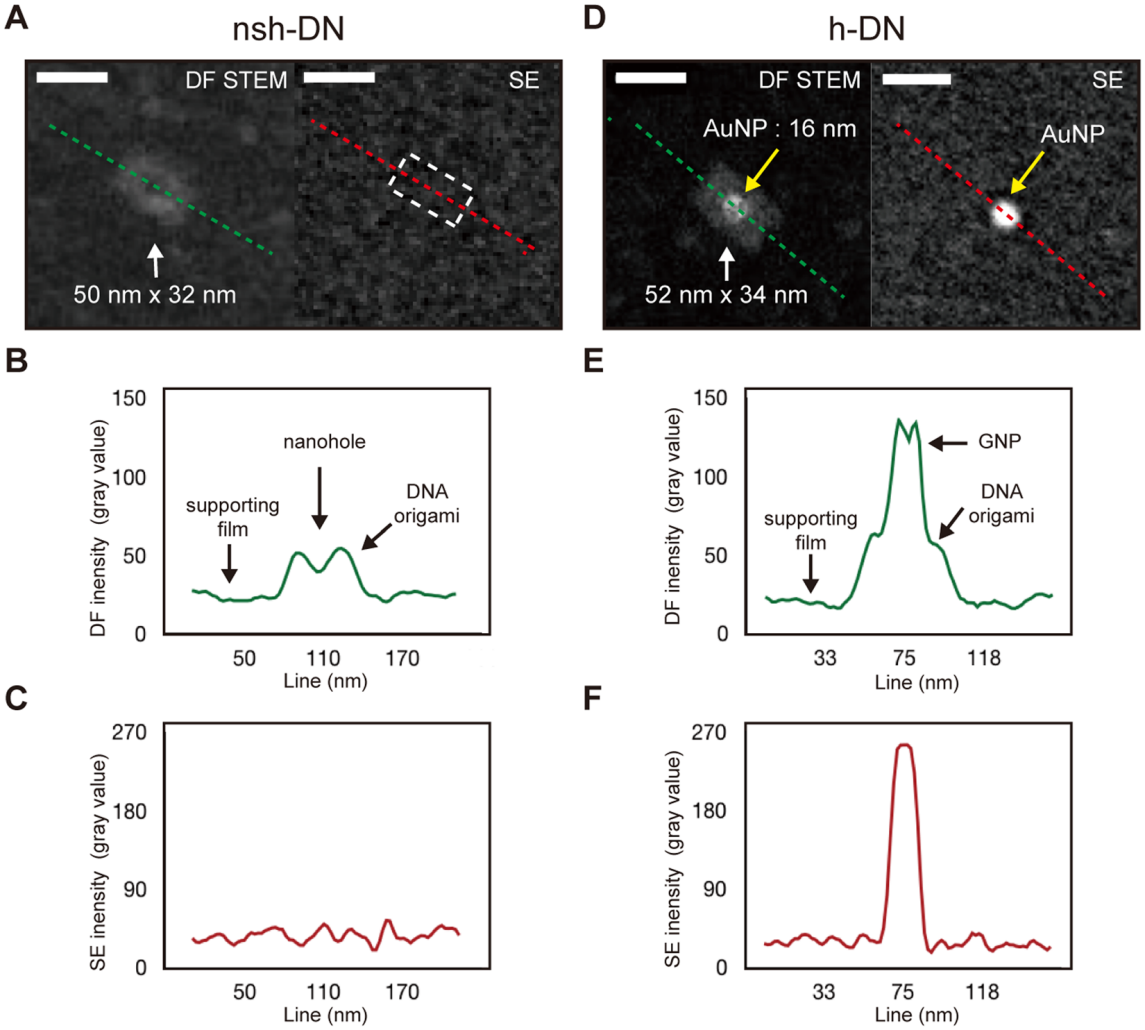
EM observation and analysis results of the nano-square holed and the heterogeneous DNA structures. (**A**) Micrographs of the nsh-DN using STEM and SE modes. The STEM image distinguishes the opening region of the nano-square hole from the rest of the DNA origami region with a contrast difference. The SE image largely fails to show the boundary, if any, of the DNA origami. The corresponding region is indicated with a dashed white box. The intensity line profiles of the STEM and SE modes were measured along the green and red lines, respectively. Scale bars are 50 nm in all images. (**B**) Line profile of the nsh-DN with the STEM mode. The DNA origami, nano-square hole, and supporting film are clearly shown via the difference of their DF intensities. (**C**) Line profile of the nsh-DN with the SE mode. DNA origami cannot be observed. (**D**) Micrographs of the heterogeneous nanostructure consisting of a DNA nanostructure and an AuNP with STEM and SE modes. The STEM image clearly reveals three regions: the DNA origami, AuNP, and supporting film. The AuNP is indicated by a yellow arrow. In the SE image, the AuNP is extremely bright whilst the DNA origami region shows no signal. (**E**) Line profile of the heterogeneous structure with the STEM mode. Three regions—AuNP, DNA origami, and supporting film—are clearly distinguishable. The DNA origami region shows a similar intensity to that of the nsh-DN in (**B**). The AuNP shows twice the intensity of the DNA origami region. (**F**) Line profile of the heterogeneous structure with the SE mode. Even though the DNA origami region provides no signal, the AuNP shows very high intensity.

Next, the nsh-DN and h-DN were evaluated. Spurred by the layered DN observations via LV-STEM, holed DN at the center were observed with and without AuNPs; Fig. 4 summarizes the observation results with the STEM and SE detectors. In Fig. 4A, the DF-STEM image shows a DNA nanostructure (nsh-DN) in bright contrast. At the center, a dark contrast region is observed. In Fig. 4B, the line profile of DF intensity along the green dotted line in the DF-STEM image shows a drop in the signal intensity at the center of the line. This region corresponds to the nano-square holed structure of the DNA origami, that is, the central opening normal to the DNA helix direction. As mentioned earlier, the AFM experiments failed to resolve this nano-square hole. (See Fig. S2 in Supporting Information section 2). The hole can be considered as a region of reduced thickness of the specimen. Therefore, DF-STEM can acquire information on the hole region in the vertical direction.

On the other hand, with the conventional SE detector of the SEM, the DNA origami was not observed in the SE images because of the low probability of the inelastic scattering that generates SE. This shows the superiority of the STEM mode in observation of DNA origami.

Figure 4D shows the observation results of the h-DNs. The DF-STEM image revealed the heterogeneity of the structures, that is, an AuNP with a circular shape was observed with bright contrast on the top of a rectangular DNA origami structure. In Fig. 4E, the line profile of DF intensity along the green dotted line in Fig. 4D clearly shows the distinguishable intensities of three regions: the AuNP at the center of the structure, the DNA origami, and the supporting film. The DNA origami region shows similar intensities in Fig. 4B and E. Therefore, it seems that STEM allows visualization of the different materials with distinct contrasts.

In the comparison of this experimental result with the theoretical performance of our LV-STEM system, we executed Monte Carlo simulations with CASINO software to replicate our experimental conditions^39^. The simulated data as shown in Fig. S5 (See Supporting Information section 5) predicted that the DNA origami region of the h-DN and the AuNPs have distinct contrasts against the 3 nm carbon supporting film. Intriguingly, the DF intensity of the AuNPs depended on their diameter; the intensity of the center region of the AuNPs become darker as the diameter increased. The simulated image of an AuNP with a diameter of 16 nm on the center of a DNA origami showed a similar DF intensity with the actual LV-DF-STEM images in Fig. 4D.

In both experimental and simulated STEM images, the DF intensity was lower than the expected value that considers the mass differences between the DNA and AuNPs. We attribute this to the characteristics of our LV-DF-STEM detector, which is optimized to detect signals from electrons scattered at low angles by light-element materials. Therefore, most of the electrons, which were scattered at higher angles by the AuNP, were not detected.

As shown in the SE image in Fig. 4D, even though the origami structure cannot be observed, the AuNP shows a very high contrast because of the high SE yield of gold compared with carbon-based soft materials. This SE mode can be regarded as a complementary method to the STEM mode with some advantages. The high signal level of the AuNP with the SE mode can easily locate the position of the h-DN in reduced imaging acquisition time compared with the STEM mode; the h-DNs can be found rapidly and imaged clearly using the SE mode.

Overall, the above observations show the effectiveness of STEM for analysis in the vertical direction of nsh-DN, while the AFM experiments failed to visualize the nsh-DN. STEM can simultaneously visualize both AuNPs and DNA structures in heterogeneous h-DNs, whilst the SE mode provides complementary information to the STEM mode and enhanced processability.

### Direct observation of the unstained DNA nanostructures with electron microscopy

It is noted that the direct observation of the unstained DNA nanostructures without averaging by electron microscopy has rarely been reported in spite of the importance. Several studies have imaged the unstained DNA specimen using advanced TEM such as aberration-corrected^40^, Cryo-^29–31^, and energy-filtered TEM^32^. The Cryo-EM has achieved a high-resolution of sub-nanometer and successfully visualized various types of DNA nanostructures in detail, but required the multiple-image acquisition and averaging process, which take the time of several days to accomplish the entire process. The Cryo-EM still has a limit in the observation of specific DNA nanostructures with the single image acquisition. Kabiri *et al*.^28^ have reported images of the unstained 2D DNA nanostructures on the graphene films with an aberration-corrected TEM at 300 kV. However, unstained nanostructures were almost buried under the background morphology of the graphene supporting film. According to our theoretical calculation (See Fig. S4 in Supporting Information section 4), the DF signal for LV-STEM at 30 kV in this study is 11 times higher than that for the conventional TEM at 300 kV. The low signal level for the TEM is attributed to small scattering cross-sections of high energy electrons to light-element materials. The observation under the high voltage condition caused the small difference in the scattering probabilities of the electrons against the specimen and supporting film. A high angle annular DF detector, which was adopted in the study^28^, further reduces the signal because most of the electrons were scattered into low angles. This explains the low signal-to-background ratio of the images of the unstained DNA origami structures in the previous study^28^. Kabiri *et al*.^41^ have pursued the issues in their succeeding study. To image the unstained DNA origami structures at a low voltage condition of 20 kV, the authors have introduced a Cc and Cs corrected TEM that eliminates the chromatic aberration and improves the resolution. The authors have reported the improved signal-to-noise ratio and the clear DNA structures with the image reconstruction based on the single-particle analysis method. However, it is still hard to recognize the DNA structures in a single acquisition image and image averaging is necessary.

In this study, the 3D-DNA nanostructures were observed with high contrast to the background with the single image acquisitions. This is attributed to the lower energy conditions of 30 keV, staining free environment, and the STEM detector optimized for low angles, which can generate high signal levels. These remarkable advantages enable to quantitative analysis of the thickness of the DNA nanostructures to be executed swiftly as evaluating and comparing the contrast ratio of the DNA nanostructures in different images.

Besides, the customized STEM detector can be applied to the commercial SEM system comparably, and similar results are available after implementing STEM detectors optimized for low angles. The STEM system in this study is much simpler and cost-effective compared to the previous studies^29–31,40,41^ using the advanced TEMs. Therefore, this method, which can be easily adopted by a number of researchers for their studies, is useful especially for research on soft materials including DNA nanotechnology.

### Perspectives for further study

Driven by short acquisition time, the LV-STEM methodology has a clear advantage in acquiring time-variant structural information, compared to conventional averaging methods such as in Cryo-EM. If a robust platform is prepared to preserve the biological environment (confining buffered liquid in a thin window such as graphene or Si_3_N_4_ membranes), the short acquisition time would be essential for observing or tracking a single DNA origami structure sequentially in time. This dynamic observation could be conducted in various conditions such as photon irradiation^42^ and gas or liquid environments^43,44^. The single-shot imaging method shown in this study, albeit for static 3D DNA structures, should be considered as the first step toward a dynamic observation in the future.

In contrast to DNA nanostructures associated with AuNPs studied here, non-metallic DNA origami heterogeneous structures such as combined DNA and protein structures^45^ have been important in DNA nanotechnology. In our previous study^35^, the LV-STEM has clearly revealed the twisted structures of insulin amyloid fibrils. Considering a protein-DNA origami heterogeneous structure, our theoretical estimation (based on a similar method in Supporting Information section 4) suggests that LV-STEM could show clear contrast differences between the DNA and protein, thereby enabling to distinguish the materialistic identity. This LV-STEM system could provide the further possibility of providing us relevant information to understand the combined status or complex interactions among the DNA origami structure, nanoparticles, proteins, and surrounded environment in nanoscale.

## Conclusion

In summary, four types of 3D DNA nanostructures were studied with the visualizing strategy of the LV-STEM. The low-energy electron probe and optimized dark-field STEM detector enabled individual unstained DNA nanostructures to be imaged with fine contrast and sharp edges with well-defined dimensions from single acquisitions without an averaging process. The LV-STEM realized the analysis of the DNA nanostructures in the vertical direction by revealing the layer difference between the d-DNs and s-DNs and the position of the opening of the nsh-DN. The LV-STEM can clarify the material difference of the DNA and AuNP regions for the h-DN and the complementary SE imaging was useful to specify the locations of the h-DNs.

This study introduces two different structural designs of DNA origami structures using a new design method: first, the control of mass thickness through orthogonal substrate stacking; second, capturing of nanoparticles via DNA hybridization. Although much more remains to be done for improving yields, this straightforward method provides an easy way of DNA nanostructure design that captures either stackable DNA nanostructures or nanoparticles by replacing a relatively small number of incumbent DNA strands. The methods could contribute to the simple and sophisticated fabrication of higher-order 3D DNA homo/hetero nanostructures.

Combined with these design techniques of DNA nanostructures, the EM imaging methodology demonstrated in this study enables rapid and simple visualization of unstained specimens with contrast analysis. The low-energy electron probe and optimized dark-field STEM detector enabled individual unstained DNA nanostructures to be clearly imaged by the single acquisition without the averaging process. In spite of some loss of resolutions compared with the conventional TEM observation with staining, this novel route may be fruitful and unique for various applications for soft materials such as in DNA nanotechnology and could be employed as a process monitor and routine process to scrutinize or certify whether DNA nanostructures are fabricated as per the specified design.

## Methods

### Preparation of the DNA origami structures on a supporting film

To observe the DNA origami structure, we used ultrathin carbon supporting films with a thickness of 3 nm (S/N: 01824, TED Pella, CA, USA), which offered a low background level in STEM images and high structural strength under plasma treatment. The film was covered on a standard TEM grid with a diameter of 3 mm. The carbon films usually have a hydrophobic surface. To increase the deposition yield of the DNA origami structures on the carbon films, the pre-treatment with O_2_ plasma was applied to enhance the hydrophilicity of the surface. The plasma irradiation time was 1 min and the power was 5 W. After the plasma treatment, solutions of the DNA origami structure were dropped onto the TEM grid and kept in air for 1 min. The remaining solution was absorbed and dried at room temperature.

### Electron microscopy imaging and analysis

Through scanning the focused electron beam on the specimens with recording the DF and SE signals, the images were acquired digitally with 640 × 480 pixels in an 8-bit resolution. Then, the regions of interest were cropped from the images and constituted the figures. For contrast analysis of the LV-DF-STEM images, signal levels were measured at the selected target regions with the ImageJ program^46^. All images in this study were obtained from single acquisitions without averaging.

### DNA oligonucleotide synthesis

Synthetic oligonucleotides were purchased from Bioneer (Daejeon, Korea) and purified by polyacrylamide gel electrophoresis (PAGE). The details can be found at www.bioneer.co.kr.

### Purification of structures

The DNA origami nanostructures were fabricated by thermal annealing, then purified by agarose gel electrophoresis. The structures were run under 1 × TAE/Mg^2+^ running buffer and a 2% agarose gel (60 V, 2 hours) and then the gel was stained with SYBR Gold (Thermo Fisher Scientific, Massachusetts, USA). Each band was characterized by using a Molecular Imager Gel Doc XR system (Bio-Rad, California, USA). To extract the structures inside of the bands, the bands were physically excised from the gel on a High-Performance 2UV Transilluminator (UVP, Jena, Germany), and spin filtered with a Freeze N Squeeze microtube (Bio-Rad, California, USA). Nanodrop 2000 (Thermo Fisher Scientific, Massachusetts, USA) was used to determine the final concentration of the purified structure.

## Supplementary information

Supplementary information.
