# Supplementary material for: Visualization of unstained homo/heterogeneous DNA nanostructures by low-voltage scanning transmission electron microscopy
Source: Sci Rep. 2020 Mar 17;10:4868. doi: 10.1038/s41598-020-61751-3 (PMC7078320; doi:10.1038/s41598-020-61751-3)
Supplement: Supplementary file 1 — Supplementary information. [file 41598_2020_61751_MOESM1_ESM.pdf]

Supporting Information for

Visualization of unstained homo/heterogeneous DNA nanostructures by low voltage scanning transmission electron microscopy

Geun Won Gang[1,2,#], Jihoon Shin[3,#], Young Heon Kim[4,5], Tai Hwan Ha[3*] & Takashi Ogawa[1*]

[1] Advanced Research Institute, Korea Research Institute of Standards and Science (KRISS), Daejeon 34113, Republic of Korea

[2] Department of Physics, Chungnam National University, Daejeon 34134, Republic of Korea

[3] BioNanotechnology Research Center, Korea Research Institute of Bioscience and Biotechnology (KRIBB), Daejeon 34141, Republic of Korea

[4] Division of Industrial Metrology, Korea Research Institute of Standards and Science (KRISS), Daejeon 34113, Republic of Korea

[5] Graduate School of Analytical Science and Technology (GRAST), Chungnam National University, Daejeon 34134, Republic of Korea

#G.W. Gang and J. Shin contributed equally.

Contents

[1] Annealing protocol of structures

[2] AFM imaging of the s-DNs and nsh-DNs

[3] TEM imaging of the DNA origami structures

[4] Theoretical calculation of the DF-STEM signals

[5] Monte Carlo simulation of STEM images of heterogeneous structure

[6] LV-STEM images of the s-DNs for statistical analysis in Figure 3

[7] LV-STEM imaging of DNA origami structures with or without a defect

[8] DNA sequence tables and maps of the s-DN and nsh-DN

[9] Purification of DNA origami nanostructures

[1] Annealing protocol of structures

All the structures used in the experiments were self-assembled by mixing scaffold strands and stoichiometric quantities of each staple strand in a physiological buffer, 1×TAE/Mg^{2+} (Tris-Acetate-EDTA [40 mM Tris, 1 mM EDTA, 22 mM $MgCl_2$]). Each of the structures (s-DN and nsh-DN) was separately annealed from 80 °C to 24 °C (ramped from 80 °C to 62 °C for 80 min, then from 61 °C to 24 °C for 36 hours).

Table S1. Details of the recipe of the DNA origami solution

Ingredient	Volume	Final concentration
10× buffer	5	1× buffer
M13mp18 scaffold (100nM)	5	10nM
Staple pool (500nM)	10	100nM
DI Water	30	
Total volume	50	

[2] AFM imaging of the s-DNs and nsh-DNs

To obtain the AFM images, 2 μL of the samples were placed on freshly cleaved mica for 30 seconds, after which 48 μL of 1 × TAE/Mg^{2+} buffer was pipetted onto the mica surface. AFM images were taken by a Digital Instruments Nanoscope 3 (Veeco Inc., New York, USA) in tapping mode under a buffer using DNP-S10 silicon nitride tips (maximum tip radius: 40 nm, Bruker, Massachusetts, USA).

The s-DNs were observed in Figure S1 under liquid conditions. The line profile shows that the length of the single DNA nanostructure is much longer than the designed value and has a blunt edge compared with the LV-DF-STEM image.

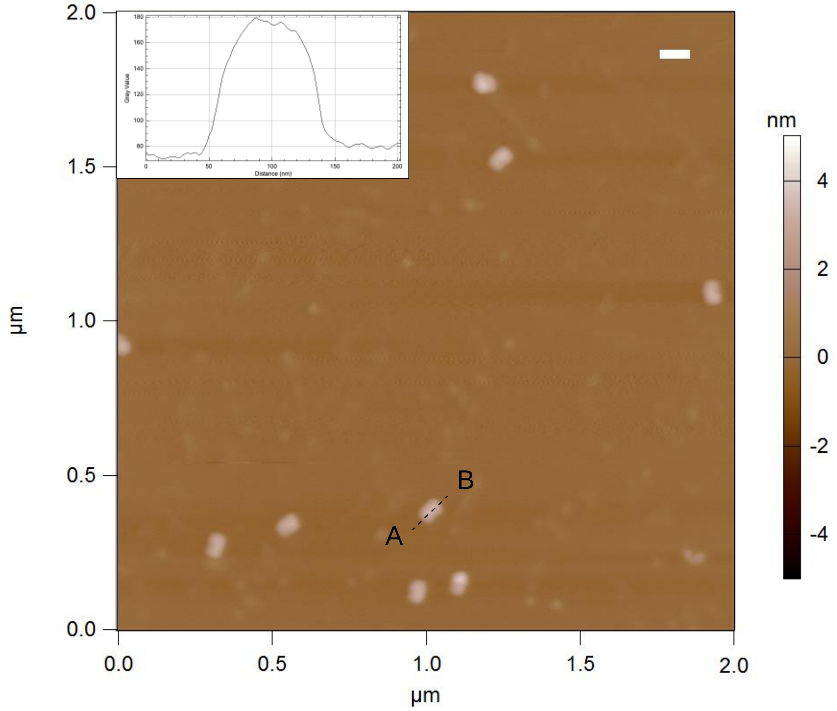

Figure S1. AFM image of the s-DNs. A line profile along the line between A and B is inserted in the top-left corners. Scale bar: 100 nm.

Next, the nsh-DNs were observed in Figure S2 under liquid conditions. As shown in Figure S2, most of the structures for nsh-DNs seem to be separated into two smaller structures with unspecific reasons, while the nsh-DNs were clearly observed as holed structures with LV-STEM observation in Fig. 4A.

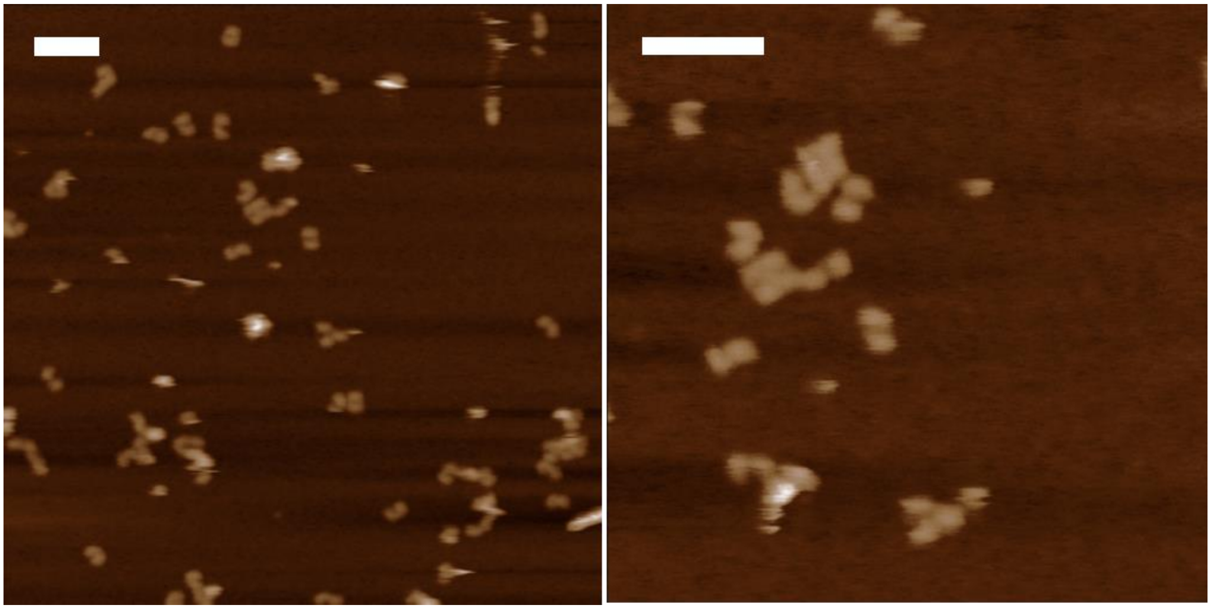

Figure S2. AFM images of the nsh-DNs. Scale bar: 200 nm.

[3] TEM imaging of the DNA origami structures

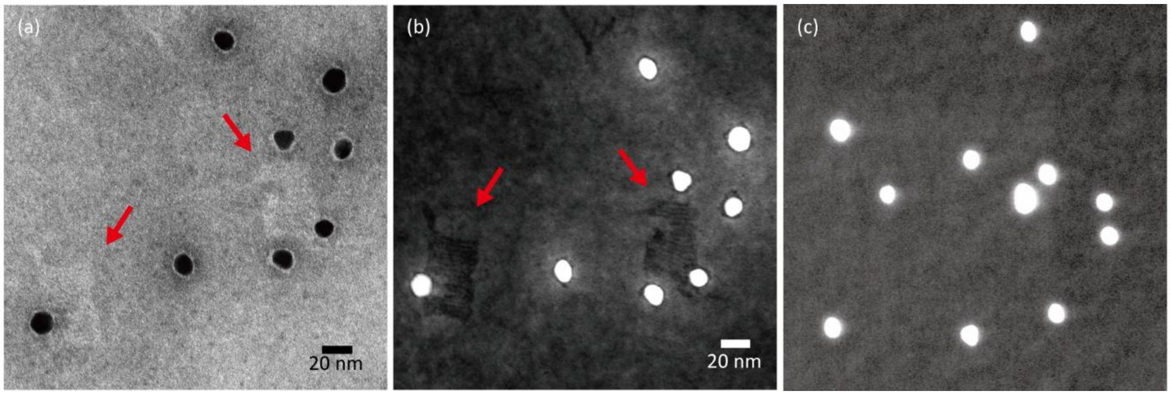

Figure S3. TEM images of the stained DNA origami structures with (a) bright-field (BF) and (b) high-angle annular dark-field (HAADF) modes. Two stained DNA origami structures are indicated by red arrows. (c) TEM image of the same specimen under the unstained environment with the HAADF mode. No DNA origami structures were found.

For the TEM observation, the DNA origami structures were prepared on a supporting 20 nm carbon film in a similar way to that described in the main text of the manuscript. TEM images were acquired by Tecnai G2 F30 (FEI company, OR, USA) with bright-field (BF) and high-angle annular dark-field (HAADF) modes at an acceleration voltage of 300 kV. The specimens including the DNA origami structures were observed under negatively stained conditions with uranyl acetate 1% solution in Figure S3 (a, b) and the same specimens were observed without staining in Figure S3 (c). AuNPs, which are sphere-like particles in the TEM images, were not combined with DNAs in this experiment but deposited for the purpose of helping TEM operation such as focusing. AuNPs of high Z element showed dark contrast with the BF mode in Figure S3 (a) but bright contrast with the HAADF mode in Figure S3 (b, c). The stained DNA origami structures were barely visible with bright and dark contrasts in Figure S3 (a) and (b), respectively. The DNA origami structures have a rectangular shape with the dimension identical with s-DN, but have a

designed opening on the one side. For unstained specimens in Figure S3 (c), we tried to observe but failed to find DNA origami structures because the DNA regions under the unstained condition did not show any contrast difference to the background of the supporting film. This is mainly attributed to the high energy condition of TEM, which causes small scattering cross-sections of electron beams to light-element materials such as DNA origami structures.

[4] Theoretical calculation of the DF-STEM signals

We theoretically estimated the DF signals of the s-DN and d-DNs to support our experimental data based on Lenz's theory,[1] which quantified the electron signals transmitted through the specimen at the specified collection angle. This equation uses a mass thickness x that is defined as $x = \rho t$, where ρ is the density and t is the thickness of a specimen. The transmitted signal $T(n_0)$ at the specific incident (scattering) angle α_0 of the electron beam is defined as

$$T(\alpha_0) = \exp\left[-\frac{x}{x_k(\alpha_0)}\right], \tag{S1}$$

where the contrast thickness x_k is given by

$$\frac{1}{x_k} = \frac{4}{Zx_{el}}\left[\frac{Z-1}{4[1+\left(\frac{\alpha_0}{\theta_0}\right)^2]} + ln\sqrt{1+(\frac{\alpha_0}{\theta_0})^2}\right]. \tag{S2}$$

Here, Z is the atomic number. The characteristic angle θ_0 is calculated by

$$\theta_0 = \lambda/2\pi R, \tag{S3}$$

With

$$R = \alpha_H \cdot Z^{-\frac{1}{3}}, \tag{S4}$$

where α_H is the Bohr radius. The transmitted signal $T(\alpha_0)$ is exponentially dependent on the value x.

Then, for estimation of the theoretical contrast of the DF images using these equations, the signals for both the DNA origami structures and supporting films are needed. Combining the

influences of the mass thicknesses of the DNA structures and films, the signal $T_{\alpha_0}^{DNA}$ from the DNA origami on the supporting films is given by

$$T_{\alpha_0}^{DNA} = \exp[-(\rho_{DNA} \times t_{DNA} + \rho_{film} \times t_{film})/x_k(\alpha_0)]. \tag{S5}$$

Meanwhile, the signal $T_{\alpha_0}^{Sup}$ from the supporting films is given by

$$T_{\alpha_0}^{Sup} = \exp[-(\rho_{film} \times t_{film})/x_k(\alpha_0)]. \tag{S6}$$

Applying the dimension of the STEM detection system to the value of α_0, the DF signals from the supporting films and DNA region are calculated by subtraction of the transmitted value at 15 mrad from that at 55 mrad as follows:

$$T_{Sup} = \left[T_{55\,mrad}^{Sup} - T_{15\,mrad}^{Sup}\right], \tag{S7}$$

$$T_{DNA} = [T_{55\,mrad}^{DNA} - T_{15\,mrad}^{DNA}]. \tag{S8}$$

The signal increase of the DNA origami structures relative to the supporting films is calculated by

$$T_{total} = [T_{55\,mrad}^{DNA} - T_{15\,mrad}^{DNA}] - [T_{55\,mrad}^{Sup} - T_{15\,mrad}^{Sup}]. \tag{S9}$$

Based on Eq. (S9), the DF signal dependence on the thickness of the DNA origami structures was derived as shown in Figure S4. Here, the thickness of the DNA origami structures was varied from 0 to 30 nm and the thickness of the carbon supporting film was 3 nm. The two acceleration voltage conditions were 30 keV and 300 keV, which corresponded to the operational condition in this study and the conventional condition of TEM.[2]

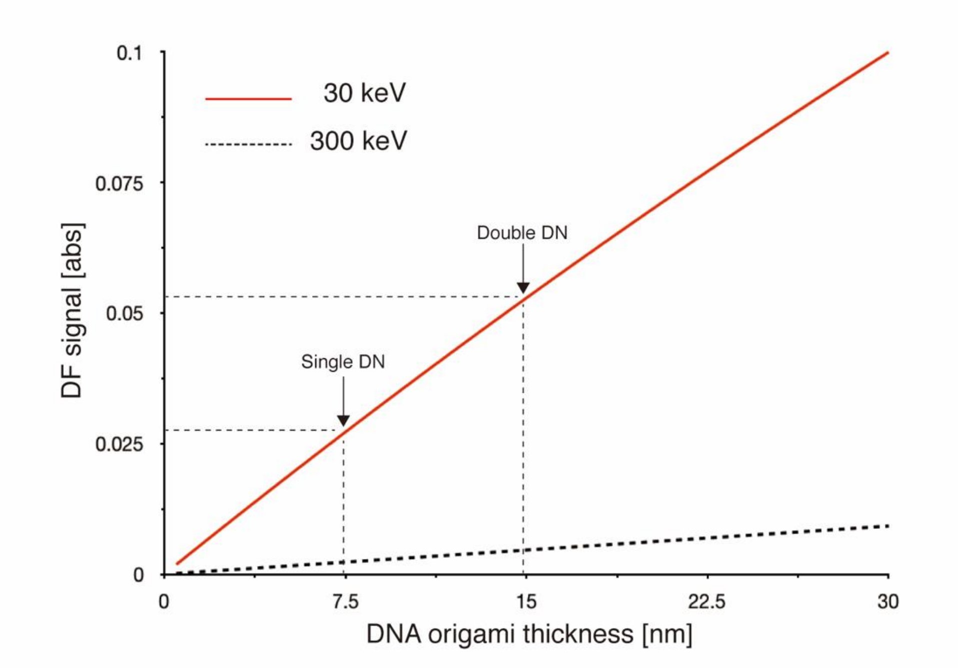

Figure S4. Dependence of the DF signal on the thickness of the DNA origami structure on the 3 nm carbon supporting film.

In Figure S4, the DF signal shows an approximately linear dependence on the thickness of the DNA origami. As designed, the s-DN and d-DN are 7.4 and 14.8 nm in thickness, respectively. For the s-DN and d-DN, the DF signal at 30 keV is 11 times higher than that at 300 keV. Therefore, under the low-voltage conditions in this study, the DNA structure without staining can be visualized in the high contrast.

We calculated the signal ratio by dividing the signal of the target by that of the s-DN. Under this definition, the d-DN and the s-DN had the signal ratios of 1.94 and 1, respectively. The two values predicted by Lenz's theory are indicated in Figure 3D with red arrows. These theoretical DF ratios are consistent with the contrast ratios evaluated from the actual LV-DF-STEM image.

[5] Monte Carlo simulation of STEM images of heterogeneous structure

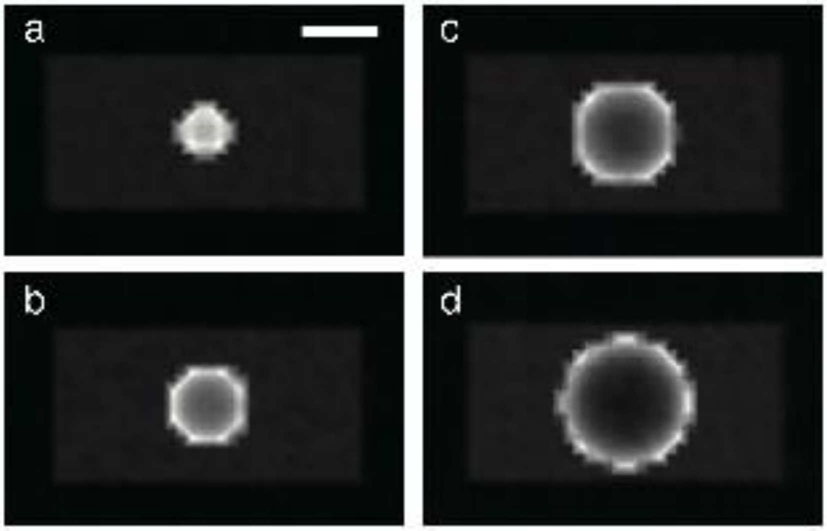

Figure S5. Simulated DF-STEM images of the heterogeneous DNA nanostructure with AuNP by a Monte Carlo method. Diameters of the AuNP are (a) 10, (b) 16, (c) 20, and (d) 25 nm. All four images have the same scale as the scale bar of 15 nm.

DF images of the h-DN containing an AuNP on the DNA origami (nsh-DN) were simulated by a Monte Carlo method (CASINO, version 3), which can calculate the electron trajectories in specimens considering physical models in 3D.[3] We adopted the same simulation conditions as our experiment, where the collection angle was 15 to 55 mrad and the acceleration voltage was 30 keV. Figure S5 shows simulated STEM images of four kinds of AuNPs of the h-DN. Three regions including the DNA origami, the AuNP, and the supporting film are distinguishable. The simulated image in Figure S5 (b) corresponds to the experimental conditions of the image in Figure 4D. As the diameter of the AuNP increases, the DF image of the AuNP becomes darker in the center region. In the radial direction, the DF images of the AuNP become darker from the outside to the

center because of the spherical shape of the AuNP, that is, the center region is thicker than the surrounding. This produces a weaker signal at the center for the STEM detector.

[6] LV-STEM images of the s-DNs for statistical analysis in Figure 3

The 110 sets of the s-DN structures have been observed to acquire the statistical data in Figure 3E and F. Figure S6 shows original LV-STEM images of the s-DNs without any image processing. The unstained s-DNs clearly show bright rectangular structures with single image acquisition. The brighter regions with white color are thicker lacy structures to support ultra-thin carbon films in dark contrast.

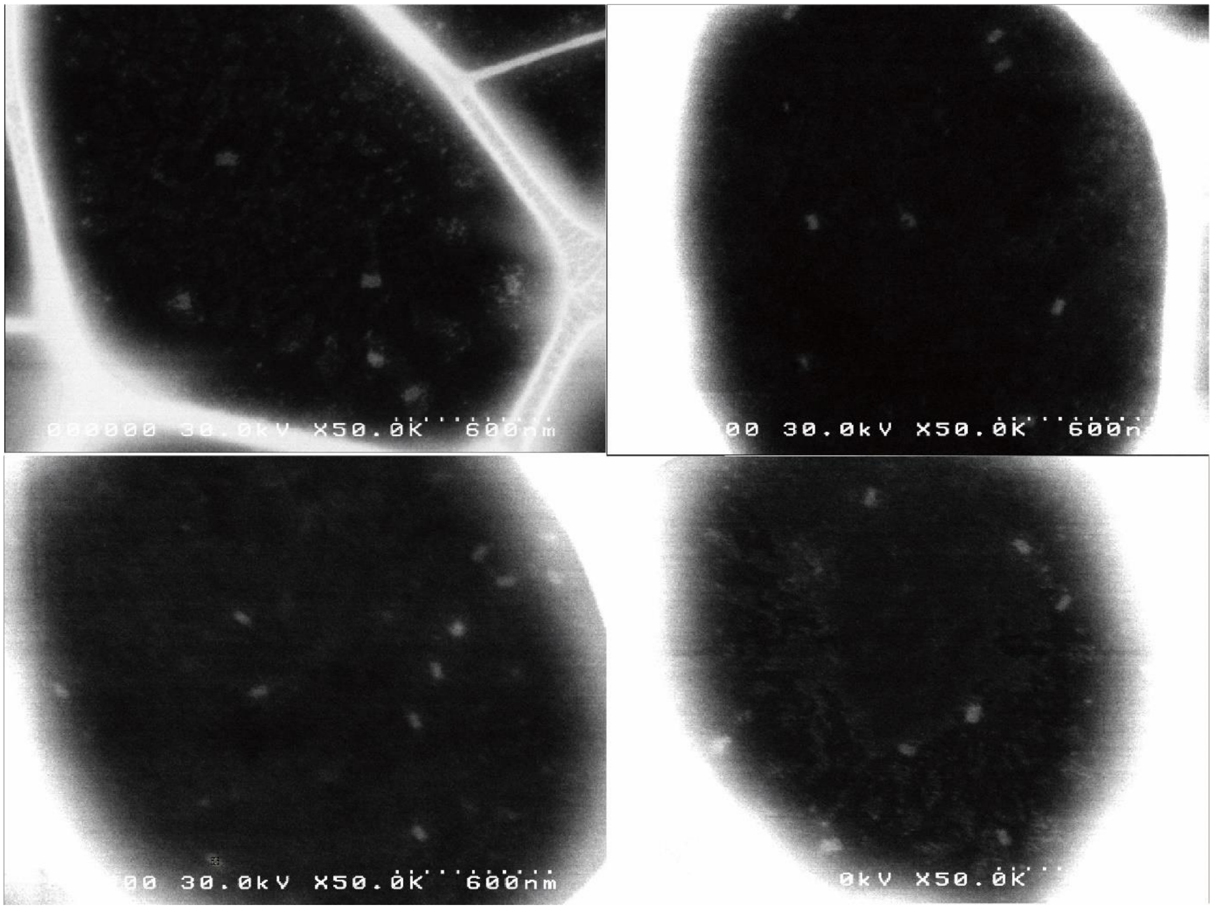

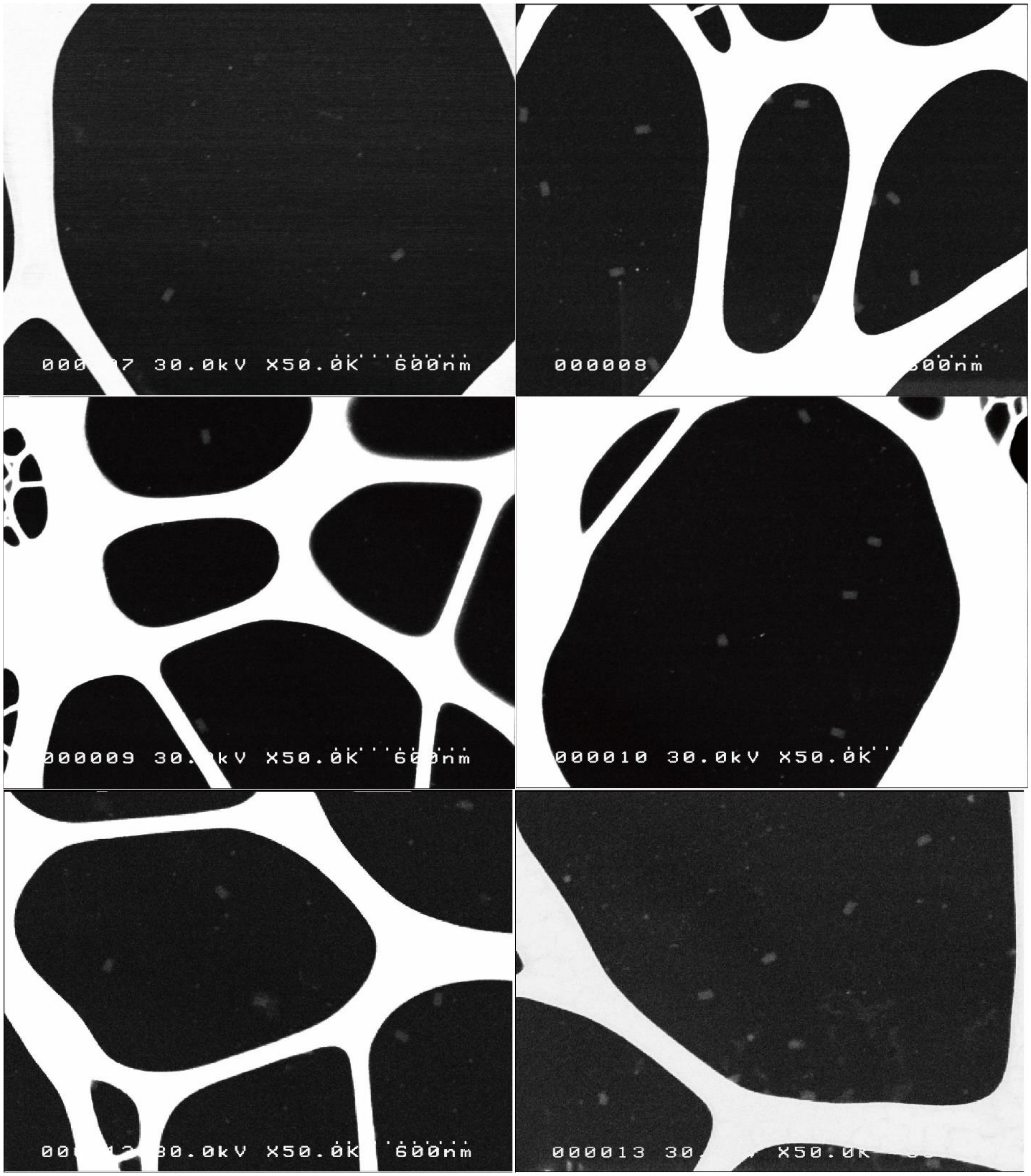
30.0kV X50.0K 600nm
000008
000009
X50.0K
000010 30.0kV X50.0K
X50.0K 600nm
000013
X50.0K

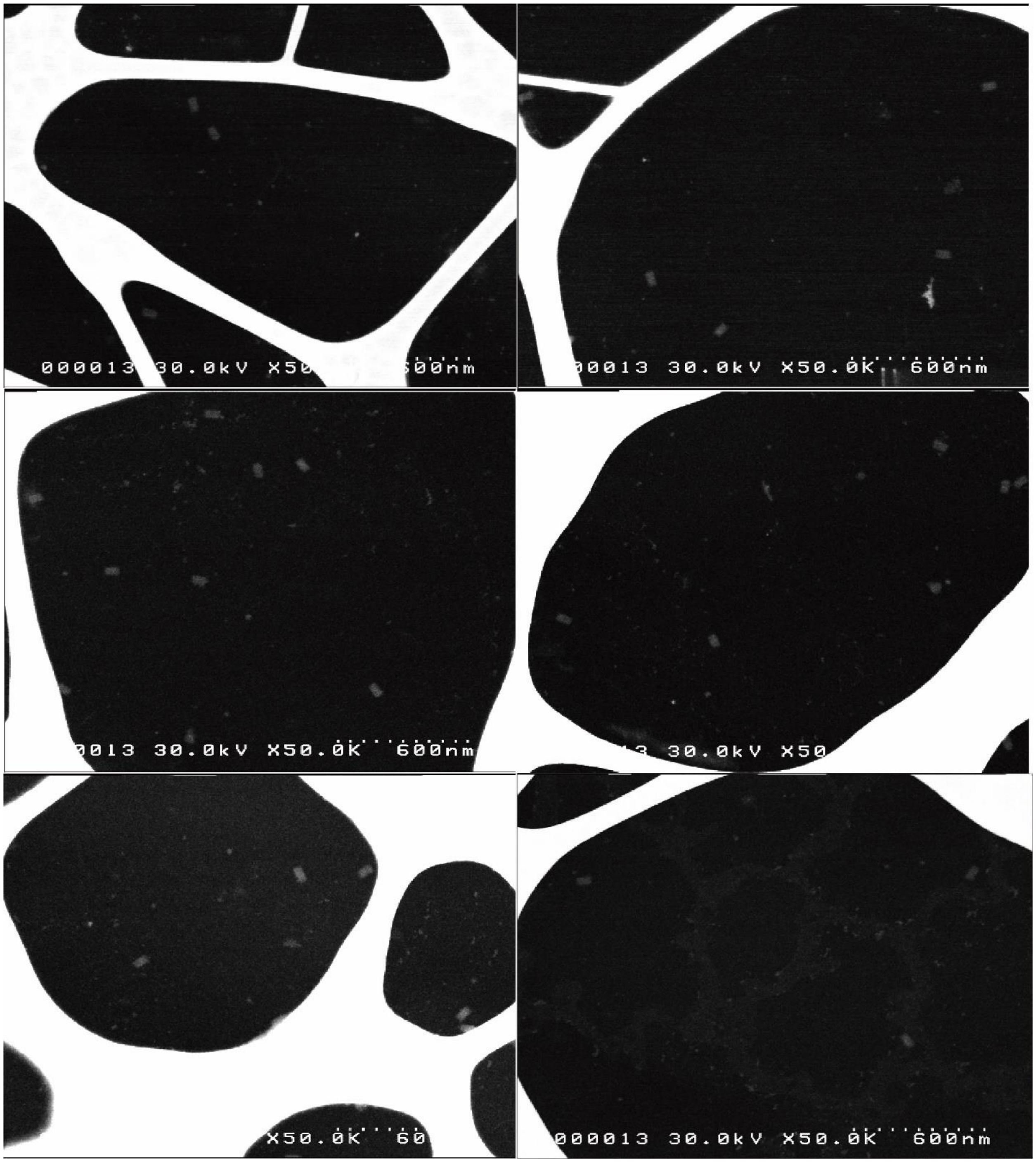
000013 30.0kV X50
00013 30.0kV X50.0K 600nm
013 30.0kV X50.0K 600nm
30.0kV X50
X50.0K 60
000013 30.0kV X50.0K 600nm

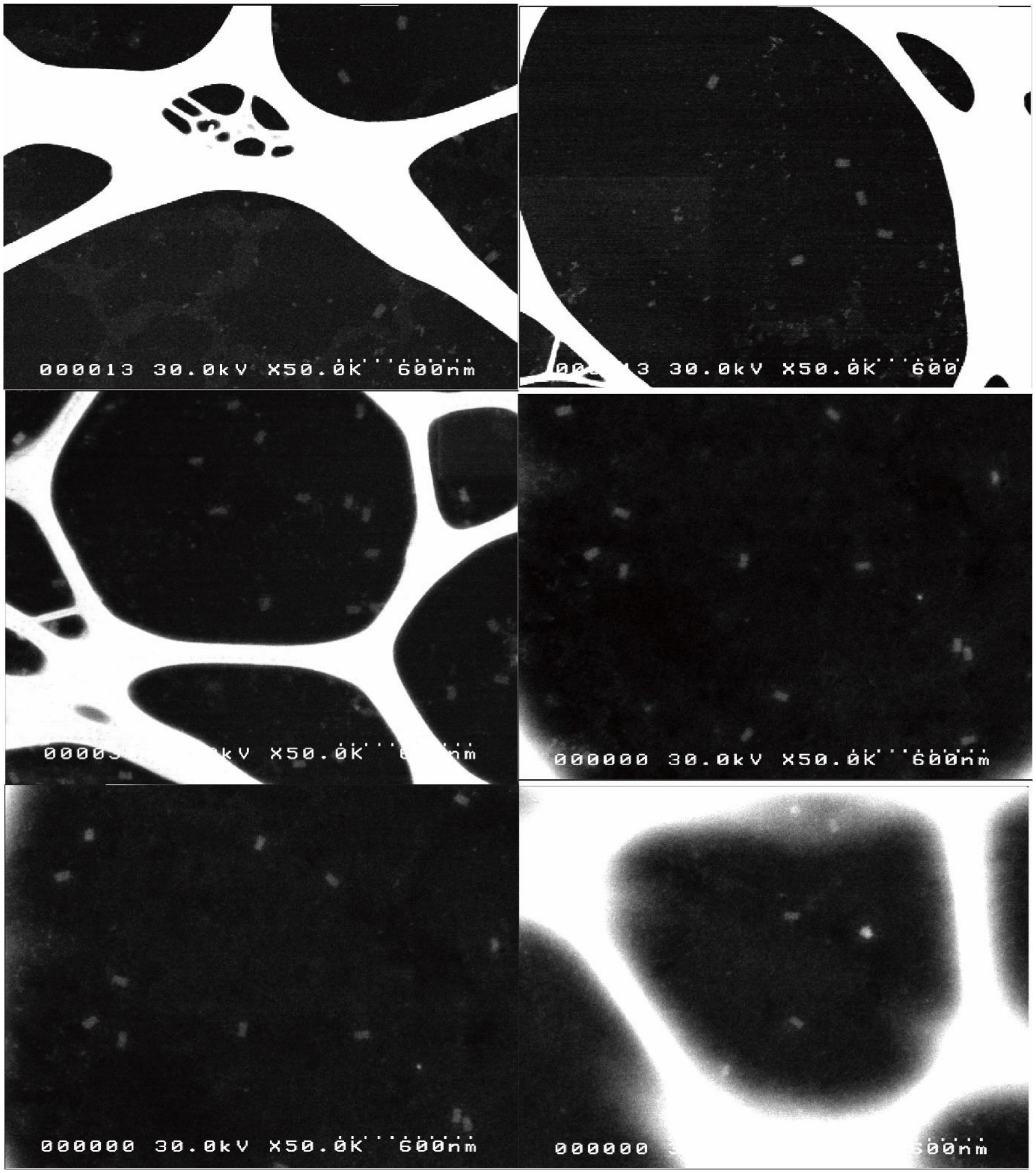

Figure S6. Original LV-STEM images of the s-DNs.

[7] LV-STEM imaging of DNA origami structures with or without a defect

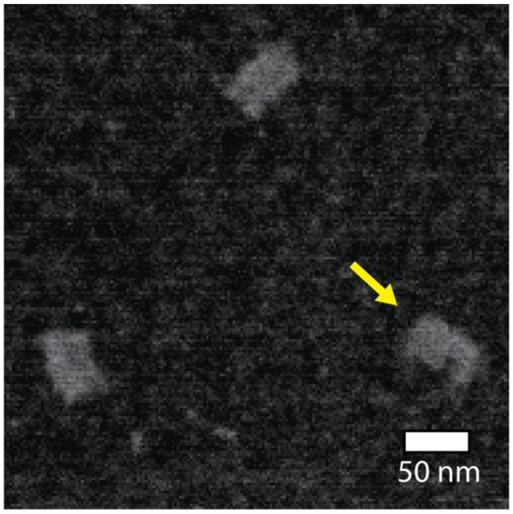

Figure S7. High magnification LV-STEM image of three DNA origami nanostructures. A DNA structure with a defect is indicated by a yellow arrow.

Figure S7 shows a high magnification LV-STEM image on three DNA nanostructures, which were fabricated in a similar process with the s-DNs. Figure S7 clearly shows the rectangular structures with sufficient contrast and well-defined edges. In addition, on the DNA structure with a yellow arrow, a box-shaped defect is recognized as a lower contrast region compared to the normal region. This result shows that LV-STEM can image not only a simple box shape, but also a non-symmetric shape of DNA origami structures, which would be more important in the field of DNA nanotechnology. The result is another evidence showing the imaging capability of the LV-STEM as a process monitor.

[8] DNA sequence tables and maps of the s-DN and nsh-DN

The sequences of all the synthetic DNA strands used in the experiments are shown below, a table listing the individual strand names, sequences, and strand lengths.

Table S2. s-DNA origami nanostructure without connection strands

Oligo NAME	SEQUENCE (5' → 3')	Length(mer)
Oligo001	TTGTAGCCTATCGGTTTTGACACCAGTAATAAAAGACGTGGCACCGAACTTTA	53
Oligo002	GTCCATCTCCAGAAACGCTCACTGGCCAACAGAGA	36
Oligo003	GGCCACCGCGCTTTCCTCGTTCAAATCAGAGTCCATGAGTGT	42
Oligo004	AAGTGTTGAGCGGGGTTGCTTGCGGTCACGCTGCGGTGGCGAAAAGGGACACC	53
Oligo005	GCTCACCGCCAACCCTCTTATCTAAAAT	28
Oligo006	ACCGCCAGAGTAAAAGAGTCT	21
Oligo007	CACGTATAACGTCAGCTAGGGCGCTGGCAAAGCGAAAAGCACTTGCAGC	49
Oligo008	TACTATGAGCTAAACCCACTAGGACTCCAAGAATAGCCC	39
Oligo009	TAGAAGCTTGCAACAGGAAAACAATATTAGGTGAGCAGTTGGCAAATTT	49
Oligo010	TAAGAATGGACATTTGGAAATACCTACACCTT	32
Oligo011	GGCCCTTCTGACCTAGATAAACTTTGCCACAA	32
Oligo012	CACGTATTAAGGTAATAACGCAAATTAACCG	31
Oligo013	ACTCGAGGTGCCGTAAGGAGC	21
Oligo014	TCGCATCACCAGAATCATTTATAATCAGTGA	31
Oligo016	AATATCATGCAACATAAAAATACAGACAATATTTTTCACACGGCTCAAT	49
Oligo017	ATCTGGTGCGGTCACAGCAGAGAAAGCG	28
Oligo018	GGAACAAAGTTTTTTGGGGAG	21
Oligo019	AGAACGTCGTGAACGAACCCTGAAAGGAAGGGAAGAAGTGTATGACGAG	49
Oligo020	ATCGAGGATTGTTTGAGATCATATTCCTGATCTGAATAAGGTTTAGAGA	49
Oligo021	GGAATTGTTTACAACGAACGTTGATGGC	28
Oligo022	TGTTCCACGGTCCACGCTGGTACGAGCCTCCCCGGCCGTGAG	42
Oligo023	GAGAGCAGGCTGGCCCTATTGGGCGCCAGGGAACCTGTCTCACATCCCC	49
Oligo024	TTCTACCTTTACATCAAGAAAAAG	24
Oligo025	GACAACTGTCAACAGTTGAAA	21
Oligo026	AAATAGAAGTCAATAACCAAAAGAATTTGAATTACCTTCTGT	42
Oligo027	AAGCGTAGCGGGGAGAGGCGGTAATGAAGGTGCCTCGCAGTG	42
Oligo028	GCCGAAAATCGCTCACATTCGTAAACGCGTGCCTGTTCGGTG	42
Oligo029	AATTCATCAATAGCTTAAATC	21

Oligo030	ACTAATCCTGATTGTACAGTAGATAGCTCCCT	32
Oligo031	GTACCTGATTTACATTTTTTAGGAAACCTCA	31
Oligo032	CAGGGAGAAAATTAGACAGGAAGGAATCAAT	31
Oligo033	ACTGTAAAGCCTGGTCGGCCA	21
Oligo034	AGTACAACATTTGCCCCATAGGGTCTATTAA	31
Oligo035	ACCTGAGGGATTCGGATTTTCATGGAAGGGTTAGAAATTATCTAACATTTCGC	53
Oligo036	GAAACAATACATCGATGAATATTTGGATTATACTTTATCAGATATTAATGAAC	53
Oligo037	TTGAGGAGGAAGCATAAAGAG	21
Oligo038	GCTCGAAATTCCACGAGCTAACGTGCCAGCTGCATTTTGCGTGAGAGAGTAAA	53
Oligo039	CATTTAATAATTTTTAGATTAATGTAAA	28
Oligo040	CCTCCTCGAGCGCGCCTGTGCTACGGCTACGTCAGTAACGGA	42
Oligo041	AAAGTCAATATAGGTTGGGTTATAAAAACTTAAGGCGTCATG	42
Oligo042	TAGTAATTACTTAGGCAGAGGCAC	24
Oligo043	AATCCTTACCAAAATTAATTA	21
Oligo044	CTGATCAGACGTTCAGCAAATCGTCTGCGGCCAATCCGTCCC	42
Oligo045	AGATCGTCGCTTAGTATTTAACAATAAAGTACCGACAATGCA	42
Oligo046	TCACTAAAAACATCCCTTACAGGTTTCTCATTGCAGGCGGCC	42
Oligo047	TGCCGGCCAGTGCCATCGGTCAGCTCCGGCCAGAGCACACGA	42
Oligo048	TGCTGATGCAAAATACATAGC	21
Oligo049	TCTCCAATCGCAAGCACCGGATATCCCAGATA	32
Oligo050	TGTGTTATACCTTGCTTTTTTAATTTCAATT	31
Oligo051	TAAAGCCTGTTATTAATCAATTTCAGATGAT	31
Oligo052	ATCATCAGCGGGGTTTGCTCG	21
Oligo053	CTTACCAGCTACTCTGTTTCGCGTGTACCGA	31
Oligo054	GCCATATCATATGCGATAAATTTTCAAATATATTTTCCGGCTGTGAATTTTGC	53
Oligo055	ATGTAATTAGAAAAGAATAAAACAAAGAACGCGAGTAACTATAGACGCTACGT	53
Oligo056	TTGTAGAGGAGGTGTCCAGCA	21
Oligo057	CTGGTCTCCACGCATCGCACTTGGTAATGGGTAAACTGGTGTGATCCAGAATG	53
Oligo058	GTAATAAAACAATATCCTAATGCAAGCC	28
Oligo059	ACGTGCCAATGATGAAGGGTAGGTGGAGATGTGCTTATTACG	42
Oligo060	GAAAAACCAATACCGCACTCATCGTAGCAAGGGTTTTGGCAA	42
Oligo061	AGTATTTTGCGATTTTTTGTTTGA	24
Oligo062	CCTGAACGGATTTTCGAGCCA	21
Oligo063	TGCAAAAAAAGTTAAATTTCTGCTCTTCTCCGTACAGCGGTAT	42
Oligo064	TAGCGCGCCTTTATCCTATCCCAATTACAGAGAGAATACAAA	42
Oligo065	TTTAGGACGCAGAAACAGCGGCTCTCACCCGGAAACACTCCA	42
Oligo066	GTATGATTGCGGCCAGTTTGGGTAGATCGGTGCGGGCCGTGC	42

Oligo067	GTTTTTATTTTCGAAAAATAA	21
Oligo068	GAATCGTAGGAATCGATTAGTGAGTTAAATCA	32
Oligo069	ACTACCAACGCAGCTAAAAGGTAAGAGAATC	31
Oligo070	CTTTACAATTGTTTATCGAGAATACGCCAAC	31
Oligo071	TGCGGATAACCTCAGGAAAAA	21
Oligo072	CGGTTTCAGAAAGTTAAATCCTCACGTGGTG	31
Oligo073	ATTATTTGAATCTTTGCGGGACAAATCAGATATAGAACCAAGTCAATAAACCG	53
Oligo074	AAGAAACACCCAGCAAATCAAATTACCGCGCCCAAAGAACAATTACGAGTAAA	53
Oligo075	AAGGGGGCCGCCACGGGAACT	21
Oligo076	GATTAAGGCCAAGCCGAAACGTGGTGAAGGGATAGATCAAACCCGCACAGGCG	53
Oligo077	GAAAATACGCTAATGCCCAATAAACCGA	28
Oligo078	CCAGCTGCCCTTTCCGGCACCAAATCAGAAAGCCCATATGTA	42
Oligo079	GTCACAATGACCGAACAAAGTTACATGATTACAAAGACTAGC	42
Oligo080	GAGCAATCAACCGACTTGAGCCAT	24
Oligo081	AGATAACGCAACGTCAAAAAT	21
Oligo082	CGGCCTCAGGACGTTGGTGTAGATTAACAACTGGCCTTTCTA	42
Oligo083	GAAAGAGGGTGGTTTACAAAGGTGACCAGTAGCACCATGTAA	42
Oligo084	GCCAGACTTGACCGTAATGGGGTGGGAACCATCAAGGAGAGG	42
Oligo085	CGGAAACCAGAATTCGCTTGTATACGTAAAACTAGCATAAGG	42
Oligo086	GGAAACGCAATAGAAAGAATT	21
Oligo087	CGATAACGGAATACTTATTTTTTTCATCCCTT	32
Oligo088	AAAAAAGACACCCTGAAACATAAACAGCCAT	31
Oligo089	CGGTTCATATAATTGAGGCAGCCTTCCAAAT	31
Oligo090	GTCCAATAGGAACGCAAACGG	21
Oligo091	AATTTTTGTTGCTTCTGTCTTCGCGCAAGGC	31
Oligo092	ATTCATTCAGCGCCAGAAACGAGACTCCTTATTACCAGATAGAATAGCACCCG	53
Oligo093	ACCGTCATAGAAAAAATAAGTCCAAAAGAACTGGCCAGAAGGAATAAGAAAGC	53
Oligo094	AATCAGACTCATTTTTTAACA	21
Oligo095	AGGAAGAATTAAATTCGCGTCCCGTCGGATTCTCCATAGGTCAAGATCGCAAT	53
Oligo096	GAGCCAGAAGTTTGGGCATTTCACCCTC	28
Oligo097	CCCCGGTCTTATTTTTGAGAGATAAAGCAAAAGGTATATTTT	42
Oligo098	TCACTTATTAAACCGCCTCCCTCACACCAGACAAATAAGTTT	42
Oligo099	TAGGCGCAGTAATGCCCCCTGCCG	24
Oligo100	CGTCAGATGATTTGGGAATTA	21
Oligo101	CTAGATAAATTTCAAAAGGGTGAGAAAATTTCTGTAATTTTT	42
Oligo102	CCCGTAGCGATTCCAGTACAGTGCCATGAAAGTATTAAACCA	42
Oligo103	GTAGCGTTGAGTAATGTGTAGTATTTTATGTACCAACGGTGT	42

Oligo104	GCTTCAGGTCAAGAATTTAATAGTTGGTCAATAACCTGGTTG	42
Oligo105	AGAACCGCCACCCCAGCGCGT	21
Oligo106	TGCTCAGAGCCACCCAGAATGGTGTATCTTGA	32
Oligo107	TATATACATGCAGCACCTACCATTGGAAATT	31
Oligo108	TCATTTACCGCAGAATCCAAAATCAATTATC	31
Oligo109	GAAGCTAAATCGGTAATGCAA	21
Oligo110	CATTTAAGCAATCTACAGTCAATCCAAAAAC	31
Oligo111	TTGAGTAAAGCGTCTCACAAAACCACCACCAGAGCCCACCGGGCGTTTGATAT	53
Oligo112	AACAGTTCTCTGAATTAAAGCACCCTCAGAGCCGCGAGCCGCTCGGTCAACCA	53
Oligo113	CGAGCTGCTCAGAGCATAAAA	21
Oligo114	ATTCTACAGCAAAATATGACCTTAGAACCCTCATAGTAAAGATAATGCCAAAT	53
Oligo115	CTATTATGAGAGGGACCGTACTAGGAAC	28
Oligo116	CATTTGGGCAGTTTCATTCCAAGTCAGATCATTGAAATACTG	42
Oligo117	GGCACCGCCATTTCAGGGATAGCACTACAACTAAATGAACGA	42
Oligo118	TATCAACTTTGGTTTATCAGCTCA	24
Oligo119	AAGTATAGGCTATTTCGGAAC	21
Oligo120	ATTTATGCAAGGTCATTTTTGCGGGGAAGCACTTCAAAAGGC	42
Oligo121	AGTGGATAAGGAGTGAGAAAGGAGGCTTGATACCGATATCGC	42
Oligo122	CTGGACCTAATTGCTCCTTTTAGGATTAATTAAGAATCATAA	42
Oligo123	AAACCCAATTGGTCTTTCTTTAAATGTTTAGACTGGATAAAC	42
Oligo124	CCATGTACCGTATTGGAATAG	21
Oligo125	CCACACTGAGTTTCATTTTGCGCAGCGACGCT	32
Oligo126	CAGAGGAACAGCTCAGTGAGGCTGCAGTGCC	31
Oligo127	TCTTTCAGCGTGCCGTCTCTGAAACCGTATA	31
Oligo128	TATGCATCAAAAAGGAGAGTA	21
Oligo129	GCCCTATTATTATAACATTTAGCTGGCATCA	31
Oligo130	GGCTCCAAATAGAAACGTTAGGCCTGTAGCATTCCCCCTCATCCCTCAGTTGA	53
Oligo131	TTGTATCCAACAGTGTATGGGGTCACCAGTACAAAAGCCCAATCAGGAGATCC	53
Oligo132	TAAATATAGCAAAGCGGATAA	21
Oligo133	TCAAATGACCCTGACGAAAGAAACTCCAACAGGTCGATAAGACTAAAGTAAAA	53
Oligo134	GAATTTCTAAAGGCAAGACAGCACTACG	28
Oligo135	CGGAATCCGGTTTACCAGACGGCTTGAGTCATTACCCAAATC	42
Oligo136	TGATAGCAACAACGGGTAAAATACCTAAAACAATTGTGTCGA	42
Oligo137	TTTTACTTAGATCTTGACAAGAAC	24
Oligo138	GCGGGATGTTGCTTTCGAGGT	21
Oligo139	CAAGTAAGAGTTGAGATTTAGGAAATCTACGTTTAAGAACTG	42
Oligo140	CTTACCGATACCGAACTTACAGACCAGGCGC	31

Oligo141	GGGGGCTTGCAGACGGTGGCTGACCTTCATC	31
Oligo142	CCCTCTCACAGGTAGAAAGATTAACGGATTGTGAATTAC	39
Oligo143	AAGGCACCAACCTTACCCTCA	21
Oligo144	TATAAAACGAAAGACTGCTCCATCAACTTTAATCAACAACAT	42
Oligo146	AATGAGGCGCAGGGAGTTTAAACACCTTTAA	31
Oligo147	CTTAAATTGGACGATAAAGCGTCCATCCCCC	31
Oligo148	ATAGGCTCAATCATCCTGATAACTCATCTTTGACCTCCATTAGGCTACATTTC	53
Oligo149	AAGAGTACCGGAACCCGCGACGGCAAAAGAATACAGTAATGCCATCGGAATTT	53
Oligo150	CGGATATATGGTTTAATTTGT	21
Oligo151	AACGTAAGAGTAGTATGCGATTTAATAAAACGAACTCATCAGCAACACTGGAA	53

Table S3. Connection strands for s-DNA origami nanostructure

Oligo Name	SEQUENCE (5' → 3')	Length (mer)
A0f	ACACTCAAAAATACTTAGGTGTGTTTCAGTCGGGTT	36
A0e	CCGGCGATGGCAGGAGGGCCAGTTTCACTTCACGGTTAAACAAT	44
A56f	TCGAAGGGAATATTCGGGTTGCTTCTATCCATCGAGACGCAGGG	44
A56e	AATAGCGAGAGGCTAGTAATTATTTAGACAACAGGTATCGC	41

Table S4. Connection strands for s-DNA origami nanostructure (counterpart)

Oligo Name	Sequence (5' → 3')	Length (mer)
B0f	GTGCCACACTCAAAAATACTTATTGTTTAACCGTGAAGTGA	41
B0e	CCGGCGATGGCAGGAGGGCCAGTTAACCCGACTGAAACACACCT	44
B56f	TCGAAGGGAATATTCGGGTTGCTTGCGATACCTGTTGTCTAAAT	44
B56e	AATAGCGAGAGGCTAGTAATTCCCTGCGTCTCGATGGATAG	41

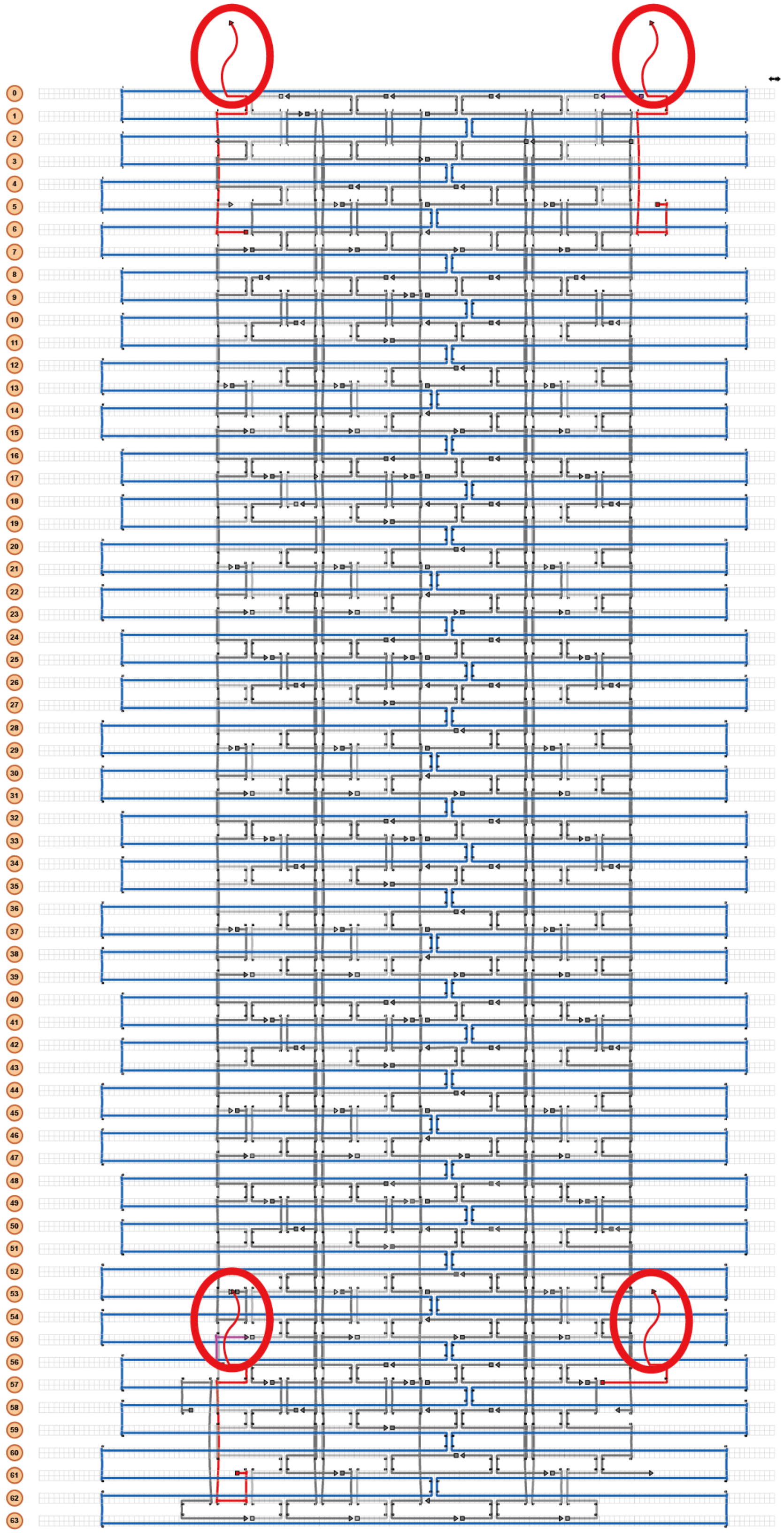

Figure S8. The sequence map of s-DN. The four single-stranded regions enclosed by the red circles in the sequence map show the connection domains of the s-DN.

Table S5. nsh-DNA origami nanostructure without connection strands

Oligo Name	Sequence (5' → 3')	Length (mer)
Oligo-h001	AGGATAACGCCTTAGCCTGATGGTAAAA	28
Oligo-h002	TTGCCAGAGGGGGTATACTGCCAAAAATCAAAGCG	35
Oligo-h003	GGAATCGTTGCTGATGGAAGTTGACCAT	28
Oligo-h004	AATTTTACGCGCGAAAGAAACAAGCAAGCCGTTTTACCGCACCATGTAG	49
Oligo-h005	AATTGCCGTTAGTTCTAAAGCATGTACCGTAACACACCCTCTACTCAG	48
Oligo-h006	GTAGCATGGTGGCAGTGAGAACAAAGGCTATC	32
Oligo-h007	AAGGGCGAGCTTTCAACGGCGAACTAGCGAGAATC	35
Oligo-h008	ACAGAGGCCCTCAGCGCATAAGTAACGAAAATGAA	35
Oligo-h009	TAGATACTATTTTCGGCAAGGCAGAGCATAAAGCTCCTTTATCCTGAGTA	50
Oligo-h010	CACCAGAGGTTTGCAATCCTGATTATCAGTTTGGAGAATAA	41
Oligo-h011	TTTTGCTTTTGCTCAGAGAAGGTGCCCGTATAAACGGTAATA	42
Oligo-h012	GAGCAAACAAATGTCAAATAAGCAAAAATTCGCATTAATAGGAAC	45
Oligo-h013	TTTTAAATATGATATTAGCTAATTTCGCTAAAGTA	35
Oligo-h014	TCCAATCGCAAGACTTAGTTACTGACCTTAAATAAGG	37
Oligo-h015	TTCGTAACTTTCCGCTCAGGACGTTGGT	28
Oligo-h016	CGGATTCCCTTTTACATCGGGGAATATATAGATTTGAGCGGAGCAATTC	49
Oligo-h017	GCTCCTTAGCTTAATCATAAAAGAAGTTTACCAGA	35
Oligo-h018	GATTGCAGAGCTTCTTTAATTTCATACA	28
Oligo-h019	ATGTGTAGACAGTGCGCGAGTTTAGTTTTCATTC	34
Oligo-h020	AATAATTTAAAGGAGAGAGACAGATCTTATTTCAGCTGTTTAATAAAGC	49
Oligo-h021	AACAAAGACGGGAGTTAACGTGTATTCTGGCGTTTAGAAAAATTTCATT	49
Oligo-h022	GTGAATTATTAAGACCTTGAAAAACCAA	28
Oligo-h023	TCGTCACTTTGAGTGCCACTACAATAA	27
Oligo-h024	CATATAAGGCTTAGTTGATAATCGCGTTTTAATTCTCAAAAAGAATGAC	49
Oligo-h025	GAATCTTTCCAAATTTTTTGTAATTAACTGCCAATCAAGCAA	42
Oligo-h026	AAAAGGATCGAGGTATTCGGTTTGCGGGA	29
Oligo-h027	ATGGCTTTCGGAACGAAACATGAAAGTAGTCGAGAATCACCGATTTTCAG	50
Oligo-h028	GCTCGAAGCTTGCAAGGGTTTTCCCAGTGCTATTA	35
Oligo-h029	ACTGGCACAAACGTCGGAATATATGGTTT	29
Oligo-h030	ATTTGGGCAAATCATAGCTATTTTTGAGA	29
Oligo-h031	GCCTGGGGCGTTGCAATCGGCGCGCCAGGGTGGTTATTGCCC	42
Oligo-h032	AAACGAATCGGACAATGACAGTACGATAAGAACTTATAAGT	41
Oligo-h033	GACAACTAAGTATTGTCAAAGGGCGAAAGTAATCTCGAGAAAGGTTTAAT	50

Oligo-h034	TTTACCGTACAGGACCATCTTCCTTTAGCGTCAGACTGTAGC	42
Oligo-h035	CCTGGCCGTATTGGCAACGCGGTGCCAA	28
Oligo-h036	GCCAAAAATCAAGAAACCGTCATTTGAG	28
Oligo-h037	GTCGGATTACCCCGCTGAGAGTCTG	25
Oligo-h038	AAGGATAAAGCCTCAAAGAAGATTAGA	27
Oligo-h039	ACCAGCGAGGGAGCCGACTTGAGCCATGAGCCAGACATACA	41
Oligo-h040	GAATTATTCATTTCGCAAAAGAAGATGATCTTTGATACGTAA	42
Oligo-h041	TCATAACACATAACAACATTAACGTTGGGAAGAAATTGAGAT	42
Oligo-h043	GACAGCGGGTAGCCTAAAACGACTAAT	27
Oligo-h044	GCTATTCCTCCCGAGAAGGCATCGTAGGAATCATAGAACGG	41
Oligo-h046	TGAATTCTTCCCCCAGTTTGAACCAGGCTCGCCATTGTGCTG	42
Oligo-h047	GCTCACTTCCGAAATGTTCCAAAATTCT	28
Oligo-h048	GATTTAGCGTATTACCGAACGATTTTGC	28
Oligo-h049	CTTATTAACCCTCAGCCGCCACCAGAACTTGGCCTCTCTGAAGAGGTTT	49
Oligo-h050	GGCGCATACGACAGTATCGGCGCACCGCTGTTGGGCGCCAGC	42
Oligo-h051	CGGGATTATCAACGTCCACTATTAA	25
Oligo-h052	TTCAACAGTCAGGTTACAGGCATAAAT	27
Oligo-h053	TTCCAGAGAATAATCGTTGAAAATCTCCGCTTGATTCGCCCACAGCGAAA	50
Oligo-h054	CCAAGTTACAAAATTTGCACGTTAGAACATATAAT	35
Oligo-h055	AGCCAGACAGAACCGCGGGGT	21
Oligo-h056	CAAAAATATTATTTATAGCTACAGAAGGCAAAAGA	35
Oligo-h057	ACCAGACTCAGAAGCAGGTCTACGGAAC	28
Oligo-h058	CAAGACTCCGAAAGTTTAAACATAACCT	28
Oligo-h059	GCGTTTGGTGTACTAGTTAATGCTGAGAC	29
Oligo-h060	GCGTCTTCAGCCATGAAAATAGGGAAGCGCATTAGTCAGAGGAATAATA	49
Oligo-h061	CGGTGTCATATAATGAGTACCAAAGCGA	28
Oligo-h062	AAAAACCGAGGCTTTTGCAAATATTCATAAAACGAGATTAAG	42
Oligo-h063	TCCTCAAGTACCAAGTACCGACCCTCAG	28
Oligo-h064	ACTATATCCTCCGGTAGGTCTGTCAATA	28
Oligo-h065	TAATGGAAAATCAATATATGTTATTAATTTAGAATCGCTGAG	42
Oligo-h066	ACAGCGAAAAATGAGCCGGAATTCCAGTGTCGTGCTCACCAGTG	44
Oligo-h067	ACGAAGGCACTAAATTAAACAAAAAAAAACTAAAGG	36
Oligo-h068	TGCGGATCAGTTGATGCGAACGAGTAGACTGAAAATAACATCTTGTACC	49
Oligo-h069	CGACGATCCACATTATTCATCTGGCTCATTATACCTTTAATCAAGGCTT	49
Oligo-h070	TTACCTTAAGAACAGTTGAGAGTTCAGTGAATCC	34

Oligo-h071	CCCCCAGCTTGCTTGCCTTTAGAGTGAGAATAGAA	35
Oligo-h072	GAATTTCACACTCATGAAACACAGAGGC	28
Oligo-h073	TCGGCTGACTTCCCTAATTATTTTTAAGTAAATG	35
Oligo-h074	GCATAAAATTCCACACAACATATGAGGACTCTAGCGCGATTA	42
Oligo-h075	TGGAAGGGTAAAACAAATTGCGCAGTAAC	29
Oligo-h076	AATCCCTATTAATTGTGCCTATGAAATTGTTATCC	35
Oligo-h077	AAATTTAAAAATCACTTAGGTTCGTCGCGAGTGAATGAATTA	42
Oligo-h078	AAAAACAGTAATACGATTCAAAAAAAAGATCAGGGTCAATTC	42
Oligo-h079	CCATCAAGTTGCGCTCTTTCAAATTTCT	28
Oligo-h081	CACCAATGAAACCAACCAGTAGCACCATATGTTAGTGATTAACCGAACAA	50
Oligo-h082	TTAAATGGCGCAACTTCTGGTGTACCGAGCTCACAGTGTAAA	42
Oligo-h083	AAGTGCCTTAAGAGGCCCCCTGCCTATTTTGATGATTCCAGT	42
Oligo-h084	GCGCAGTTGATATTCACAAACCCTCAGAGAACCGC	35
Oligo-h085	CCTTTTTATAAGTCTCAATAAGTATTAAACCAAGTTATTTTCTTATCCG	49
Oligo-h086	ATTTAGCCATCGCTAGAAACACGCTG	26
Oligo-h087	GATCTAAGGCCGGAGGTAAATTTTGCGGGAGAAGAAATCGGCAATAAA	48
Oligo-h088	ATAGGGTTGAGTGTTCGGCAAGTTTGCCTTCACCG	35
Oligo-h089	CTGAACATAGCGAATTGCACCCTAACGA	28
Oligo-h090	AAAAACAGCAGCCTCAGATATACTTGCGACAATAG	35
Oligo-h091	ATGGAAAAGTTTTATAGCCCCGCGTTTT	28
Oligo-h092	ACAGCTGTTTCTTTCAGCTGCAGGTCGATCCCCGG	35
Oligo-h093	GACTAAAGACTTTTGCCGCTTCGCTGAGCAGACAG	35
Oligo-h094	AATGCCGGAGAGGGCCATCAATGCAATGTTCAACGC	36
Oligo-h095	AGCCACCTGAGTTAGTTAGCCCGATAT	27
Oligo-h096	GCTGTAGGCGTCCAAATAGTAAACACTA	28
Oligo-h097	CCACCACATTATCTTATTAAGCCCTGATGACAAGTGCAGATCCTCGTT	48
Oligo-h098	CCAGCAGCGGAATCATGCGTTATATATTAAAGAACTTATATA	42
Oligo-h099	GATTTTATGCAGAAAGCTGCTCATTCTACCCAAAGGAATA	40
Oligo-h100	TGGCGAATAACGCCTGCCTGCATTAATG	28
Oligo-h102	GCCGGAAGGGGACGCGTAACCGTGCATCTAAACCCGTAACCTCATTTTT	49
Oligo-h103	CCTGATTGATGATGATTATCACAAAGAAA	29
Oligo-h104	GCGAAAATGGTGGTGCCCCGCT	21
Oligo-h105	GAAGCCCAAATAAATCCAGAGGATTAGTT	29
Oligo-h106	AGTTGGGAGGGGGATCAGGCTTGAGCGA	28
Oligo-h107	CGCCAGAGGTTGAAAGCGTCTAGGTGTGGGTTGA	34

Oligo-h108	ACCCTTTATGCTAATAGTATTGCCATAAAGCTTCCCTCAA	40
Oligo-h109	CCCGGAAATCATATGGCAAACCACCGGAGTAGCGACAGA	39
Oligo-h110	TATGGGACCCTCATTCGTCACCAGTACAAACCGCCCCACCCT	42
Oligo-h111	ACGCAAAAGAGCAAAATAGCAATCCCAAACCAACGCAGCTAC	42
Oligo-h112	GGTGACCCGAACAATCATATTTCAAAGAGAAATCATTGAATA	42
Oligo-h113	GGATAGAGGAACCTTTTGTCACAACCAACCGATA	34
Oligo-h114	GGACAGGCATTGCGGAGCCACCACCGGATCAAAAT	35
Oligo-h115	GACACCAAGAAAATCAAAATCTCGATAGCAGC	32
Oligo-h116	GAATACCAAACCGAGGAAACGATAATGAGAAACAGCACCCTG	42
Oligo-h117	ACCGTAATCAACAATTATTGGGCAACATTCAACCGATTGCCAAAG	45
Oligo-h118	CAATTCTCTATTATAATAACGTAAAGGTAAAAGAAACAAAAG	42
Oligo-h119	GGCGGATTTTTCTGAGGAACAAGGCTCC	28
Oligo-h120	TAACCAAATTTTTGAACAGGAAGATTGTTCATATGTCTCCGT	42
Oligo-h121	TAGAAAGCAACTAAAACCGGACTCCAAC	28
Oligo-h122	AGGTCATTGCGTTGATACCCCAAATTAAATCAG	33
Oligo-h123	AGGAAGCTCAAATAGAGGTCA	21
Oligo-h124	GGCAGGTCAGACGACACCACCCCCTCAGAGCCGCC	35
Oligo-h125	ATCAAGTTTGTTCATAAACCGCCTAGAGCCGC	32
Oligo-h126	AGTTACTCTTACCTAAGCCCGTAATTGAGCGCTA	34
Oligo-h127	TACCAGTGTATCATATAATTATTAAATAAGAATAACGTGTGA	42
Oligo-h128	AATTATCACCGTCAGGAAGGTAAATATTAAATTCAAGTTTATATTGAGT	49
Oligo-h129	GTAGATGGGGAACAATCAACAGCCATCAAAAATAATTTTGTTAATATTT	49

Table S6. Capturing strands for nsh-DNA origami nanostructure

Oligo Name	Sequence(5' $\rightarrow$ 3')	Length (mer)
1-AuNP	ATCTACCTCACACCACACTTCTTAGAAAACAAGAATACACCAACAACGGCT	51
1-AuNP-rm	AACATCA	7
2-AuNP	ATCTACCTCACACCACACTTCTTTTAACAATAATATCTTACGAGTCATCGAG	54
2-AuNP-rm	GAGGCAAAAAATTAATTACAT	23
3-AuNP	TAGAA AGCAACTAAAACCGGACTCCATTATCTACCTCACACCACACTTC	56
3-AuNP-rm	AAC	3
4-AuNP	ATCTACCTCACACCACACTTCTTCAAGAGTATTTGATCCTGACACGGGCA	50
4-AuNP-rm	GTTTGGAA	8

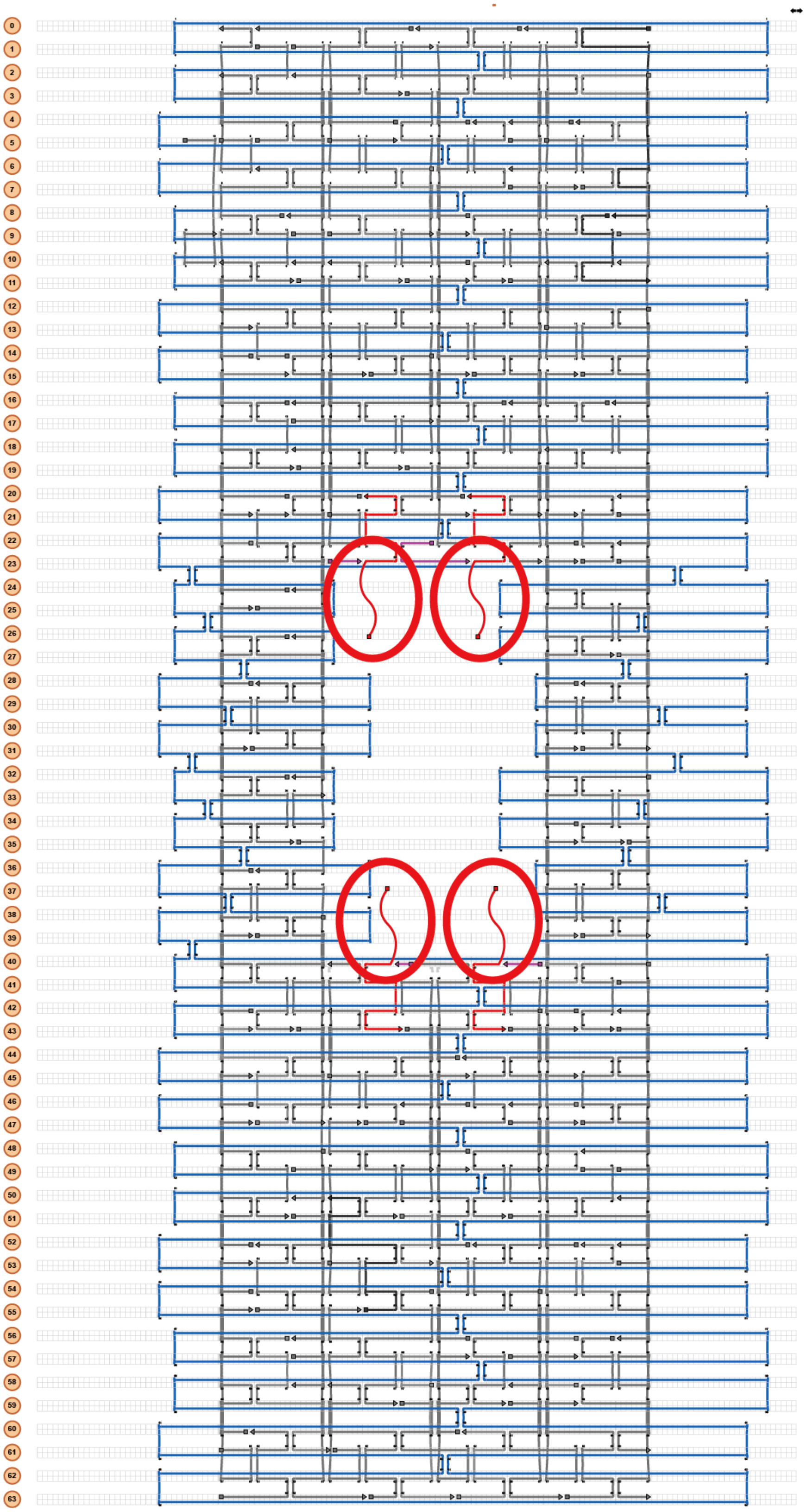

Figure S9. The sequence map of nsh-DN. The four single-stranded regions enclosed by the red circles in the sequence map show the AuNP-capturing domains of the s-DN.

[9] Purification of DNA origami nanostructures

The structures were run under 1 × TAE/Mg^{2+} running buffer and a 2% agarose gel at 60 V for 2 hours and then the gel was stained with SYBR Gold (Thermo Fisher Scientific, Massachusetts, USA) Each the bands were characterized by using a Molecular Imager Gel Doc XR system (Bio-Rad, California, USA). To extract the structures inside of the bands, the bands were physically excised on a High-Performance 2UV Transilluminator (UVP, Jena, Germany), and spin filtered with a Freeze N Squeeze microtube (Bio-Rad, California, USA). The DNA concentration of the purified structures was measured by a Nanodrop 2000 (Thermo Fisher Scientific, Massachusetts, USA). Figure S10 (Lane 1, 2, 3) shows the agarose gel electrophoresis data, which is the origin of Figure 1B in the main text. Figure S11 shows the gel image on a High-Performance 2UV Transilluminator.

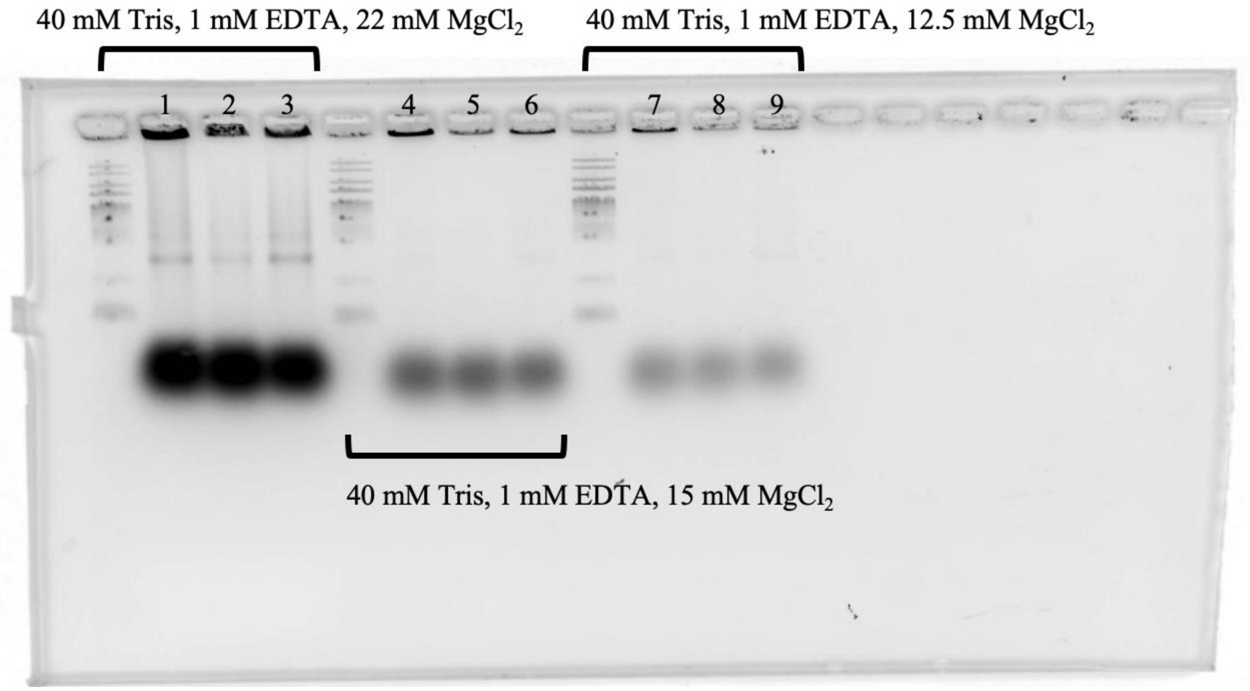

Figure S10. The Agarose gel electrophoresis data of the DNA origami nanostructures (s-DN(Lane 1, 4, 7), counterpart of the s-DN(Lane 2, 5, 8), and nsh-DN(Lane 3, 6, 9)) which is annealed with three different $MgCl_2$ concentrations: Lane 1, 2, 3 for 40 mM Tris, 1 mM EDTA, 22 mM $MgCl_2$;

Lane 4, 5, 6 for 40 mM Tris, 1 mM EDTA, 15 mM $MgCl_2$; Lane 7, 8, 9 for 1 mM EDTA, 12.5 mM $MgCl_2$.

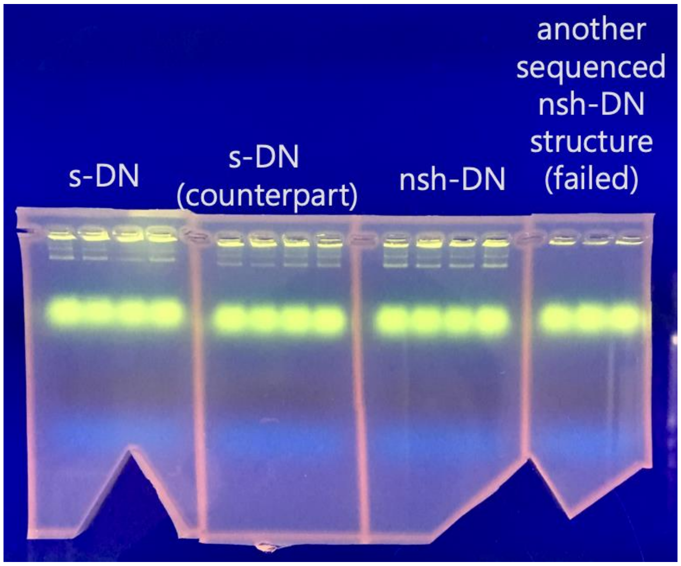

Figure S11. The gel image on a High-Performance 2UV Transilluminator. The top indexes of the gel indicate the corresponding DNA origami nanostructures.

References

1. Reimer, L.; Kohl, H. Transmission Electron Microscopy: Physics of Image Formation; 5th ed.; Springer Series in Optical Sciences: New York, 2008; p. 196.
2. Kabiri, Y., Ananth, A. N., Torre, J. Van Der, Katan, A. & Hong, J. Distortion of DNA Origami on Graphene Imaged with Advanced TEM Techniques. 1700876, 1–8 (2017).
3. Demers, H. et al. Three-dimensional electron microscopy simulation with the CASINO Monte Carlo software. Scanning 33, 135–146 (2011).
